# The FGF, TGFβ and WNT axis Modulate Self-renewal of Human SIX2^+^ Urine Derived Renal Progenitor Cells

**DOI:** 10.1038/s41598-020-57723-2

**Published:** 2020-01-20

**Authors:** Md Shaifur Rahman, Wasco Wruck, Lucas-Sebastian Spitzhorn, Lisa Nguyen, Martina Bohndorf, Soraia Martins, Fatima Asar, Audrey Ncube, Lars Erichsen, Nina Graffmann, James Adjaye

**Affiliations:** Institute for Stem Cell Research and Regenerative Medicine, Medical Faculty, Heinrich Heine University Düsseldorf, 40225 Düsseldorf, Germany

**Keywords:** Mesenchymal stem cells, Mesenchymal stem cells, Mesenchymal stem cells

## Abstract

Human urine is a non-invasive source of renal stem cells with regeneration potential. Urine-derived renal progenitor cells were isolated from 10 individuals of both genders and distinct ages. These renal progenitors express pluripotency-associated proteins- TRA-1-60, TRA-1-81, SSEA4, C-KIT and CD133, as well as the renal stem cell markers -SIX2, CITED1, WT1, CD24 and CD106. The transcriptomes of all SIX2^+^ renal progenitors clustered together, and distinct from the human kidney biopsy-derived epithelial proximal cells (hREPCs). Stimulation of the urine-derived renal progenitor cells (UdRPCs) with the GSK3β-inhibitor (CHIR99021) induced differentiation. Transcriptome and KEGG pathway analysis revealed upregulation of WNT-associated genes- *AXIN2*, *JUN* and *NKD1*. Protein interaction network identified JUN- a downstream target of the WNT pathway in association with STAT3, ATF2 and MAPK1 as a putative negative regulator of self-renewal. Furthermore, like pluripotent stem cells, self-renewal is maintained by FGF2-driven TGFβ-SMAD2/3 pathway. The urine-derived renal progenitor cells and the data presented should lay the foundation for studying nephrogenesis in human.

## Introduction

According to the International Society of Nephrology, more than 850 million people worldwide are afflicted with kidney diseases^1^, which raises the quest for alternative therapies to overcome the limitations associated with current treatments including transplantation and dialysis. One of the most promising options is the utilization of renal stem cells for treating of kidney diseases, disease modelling, and drug development^2,3^. Renal stem/progenitor cells are self-renewing, multipotent cells with the ability to generate various cell types of the kidney to maintain renal function^4^. These progenitors are in abundance during fetal kidney development in which the renal progenitor surface marker CD24 and stem cell self-renewal marker CD133 cells are required for primordial nephrogenesis^5,6^. However, in adults, CD24, CD133 (Prominin-1) and vascular cell adhesion molecule 1 (CD106)-positive renal progenitors are present in renal tubules and capsules^7^. Two progenitor cell populations can be distinguished based on the expression of CD106. For instance, CD24^+^CD133^+^CD106^−^ progenitors are present in proximal tubules whereas CD24^+^CD133^+^CD106^+^ cells are localized in the Bowman’s capsule. The latter can differentiate into a variety of cell types of renal tissue such as podocytes and tubular epithelial cells^4–7^.

Several groups have identified urine as a non-invasive and repetitive source of renal progenitor cells^8,9^. It has been estimated that each day approximately 2,000 to 7,000 cells composed of differentiated epithelial cells, bi-potential epithelial cells (transitional cells), multipotent mesenchymal stem cells, and glomerular parietal cells are flushed out from the renal tubular network and the upper urinary tract into urine^10–12^. A subpopulation of these urine-derived cells are renal stem/progenitor cells which express master renal markers such as Sine Oculis Homeobox Homolog 2 (SIX2), Cbp/P300 Interacting Transactivator With Glu/Asp Rich Carboxy-Terminal Domain 1 (CITED1) and Wilms’ Tumor 1 (WT1)^13–15^ and CD24 and CD106^16^. Interestingly, these cells exhibit stem cell properties, i.e. expression of pluripotency-associated markers such as TRA-1-60, TRA-1-81, C-KIT (CD117), CD133 and SSEA4 and possess high proliferation capacity as they show telomerase activity. Further, they endow multi-differentiation potential and like bone marrow derived mesenchymal stem cells express Vimentin, CD105, CD90, CD73 and not the hematopoietic stem cell markers- CD14, CD31, CD34 and CD45^17,18^. Although, research interest on urine derived renal stem cells is gradually increasing but the mechanistic role of genetic factors in these cells *in vitro* regarding progenitor/differentiated status maintenance is not clear.

Studies in mice have shown that Odd-skipped related 1 (Osr1), Six2, Wnt, Cited1 and Wt1 are required to maintain renal progenitor cells during kidney organogenesis^19–25^. Additionally, signalling pathways such as Fgf, Tgfβ and Notch play major roles in renal stem cell maintenance and differentiation^26–29^. The transcription factor Osr1 is an early marker specific for the intermediate mesenchyme (IM); *Osr1* knockout mice lack renal structures due to the failure to form the IM^30^. The homeodomain transcriptional regulator Six2 is expressed in the cap mesenchyme (CM) originating from metanephric mesenchyme. Six2 positive populations can generate all cell types of the main body of the nephron^31^. Inactivation of Six2 results in premature and ectopic renal vesicles, leading to a reduced number of nephrons and to renal hypoplasia^32^. Mechanistically, Osr1 plays a crucial role in Six2-dependent maintenance of mouse nephron progenitors by antagonizing Wnt-directed differentiation, whereas Wt1 maintains self-renewal by modulating Fgf signals^22,23^. Cited1 has been reported to be co-expressed with a fraction of Six2^+^ cells undergoing self-renewal and these can be differentiated in response to activated WNT signaling during kidney development^25^. Furthermore, it has been demonstrated in mice that Bmp7 promotes proliferation of nephron progenitor cells via a Jnk-dependent mechanism involving phosphorylation of Jun and Atf2^33^.

To date, research related to transcriptional regulatory control of mammalian nephrogenesis has been limited to the mouse^19,26^ or to transcriptome “snapshots” in human^13^. A recent study demonstrated conserved and divergent genes associated with human and mouse kidney organogenesis^34^, thus further highlighting the need for primary human renal stem cell models to better dissect nephrogenesis at the molecular level. Furthermore, species differences need to be considered, for example, mammalian nephrons arise from a limited nephron progenitor pool through a reiterative inductive process extending over days (mouse) or weeks (human) of kidney development^35^. Human kidney development initiates around 4 weeks of gestation and ends around 34–37 weeks of gestation. At the anatomical level, human and mouse kidney development differ in timing, scale, and global features such as lobe formation and progenitor niche organization^34–36^. These are all further evidence in support of the need of a reliable and robust human renal cell culture model.

Expression of pluripotency-associated proteins has enabled rapid reprogramming of urine derived mesenchymal and epithelial cells into induced pluripotent stem cells (iPSCs)^37–41^. Differentiation protocols for generating kidney-associated cell types from human pluripotent stem cells have mimicked normal kidney development^28,42–44^. For example, WNT activation using a GSK3β inhibitor (CHIR99021), FGF9, Activin A, Retinoic acid (RA) and BMP7 as instructive signals have been employed to derive functional podocytes, proximal renal tubules, and glomeruli^29,45–49^. Despite these efforts and achievements, there will always be variabilities between differentiation protocols, the maturation state of the differentiated renal cells and genes associated with temporal maturation during human kidney organoids formation from human iPSCs^50,51^. We propose that using native renal stem cells isolated directly from urine will circumvent most of the shortfalls and deficiencies associated with human pluripotent stem cell-based models.

Here we provide for the first time the full characterisation of renal progenitors at the transcriptome, secretome and cellular level, which has led to the identification of a gene regulatory network and associated signalling pathways that maintain their self-renewal. We anticipate that our data will enhance our meagre understanding of the properties of urine-derived renal stem cells, and enable the generation of renal disease models *in vitro* and eventually kidney-associated regenerative therapies.

## Results

### Urine-derived renal progenitors express a subset of pluripotent stem cell-associated markers and possess features typical of bone marrow-derived MSC

**Figure 1:**
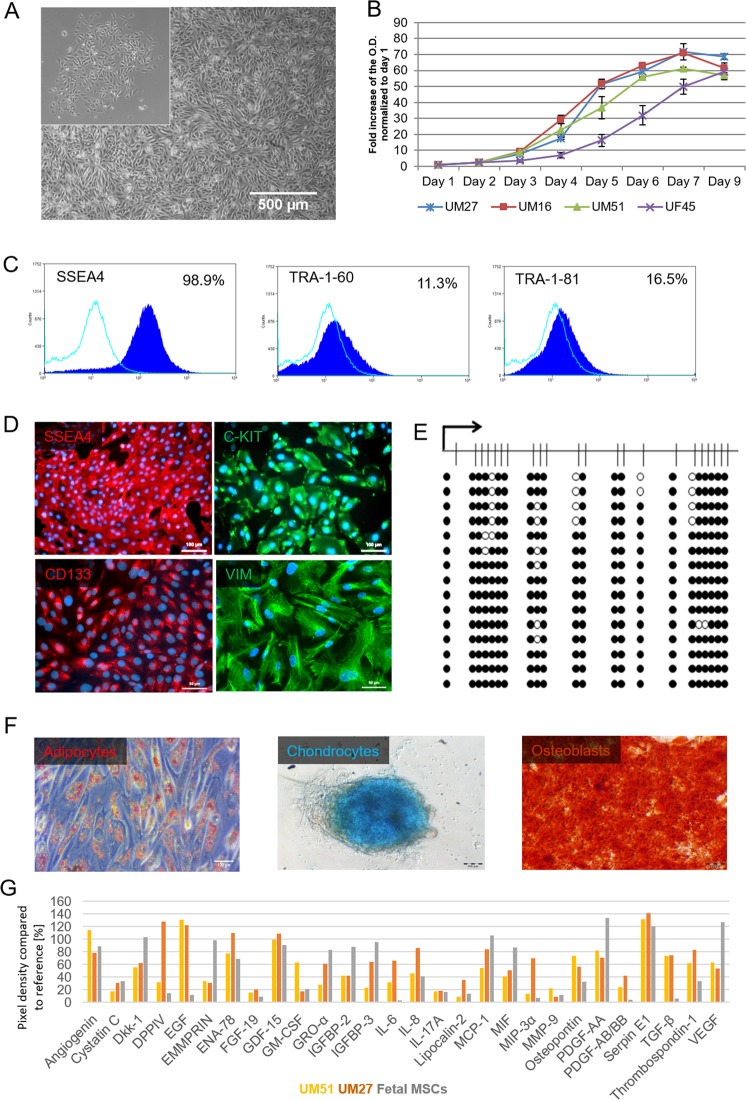
Propagation and characterisation of urine-derived renal progenitors. **(A)** Representative pictures of the “rice grain”-like appearance of the cells from the initial attachment to an elongated MSC-like morphology. **(B)** Growth curve analysis of selected urine-derived renal progenitors carried out using the Resazurin metabolic assay. Data are presented as means ± SEMs. **(C)** Immune-phenotyping for SSEA4, TRA-1-81 and TRA-1-60; and **(D)** immunofluorescence-based detection of the expression of pluripotency-associated stem cell- proteins SSEA4 (red), C-KIT (green), CD133 (red) and the mesenchymal-associated protein Vimentin (green); cell nuclei were stained using Hoechst/DAPI (scale bars: 100 µm and 50 µm). **(E)** Bisulfite sequencing of CpG island methylation patterns within the 5′- regulatory region of the OCT4 gene in UM51. Filled circles stand for methylated CpG dinucleotides. White circles stand for unmethylated CpGs. Arrows indicate the transcription start site. **(F)** *In vitro* Osteoblast, Chondrocyte and Adipocyte differentiation potential of urine-derived renal progenitors. **(G)** Cytokines secreted by urine-derived renal progenitors in culture media. Lists of significant GOs and KEGG pathways associated with the genes encoding the secreted cytokines are shown in Supplemental Fig. S1G.

Urine samples were collected from 10 healthy adult donors (4 males-UM and 6 females-UF) with ages ranging from 21 to 61 years, and of mixed ethnicity (3 Africans and 7 Caucasians) (Supplemental Table S1). Attached cells emerged from processed urine as isolated clusters after 7 days, thereafter these acquired a “rice grain” fibroblast-like morphology resembling MSCs (Fig. 1A, Supplemental Fig. S1A). A selection of distinct urine-derived renal stem cells populations (n = 4) were used to assay cell proliferation and growth. After 3 days in culture, the cells exited the lag phase and growth began in an exponential phase. Cells attained stationary phase at day 7 of subculture (Fig. 1B). All four populations- UM27, UM16, UM51 and UF45 showed similar proliferation and growth patterns.

Flow cytometry analysis revealed that approximately 98.9% of the cells express SSEA4, TRA-1–60 (11.3%) and TRA-1-81 (16.5%) (Fig. 1C). These data were confirmed by immunofluorescent-based staining of SSEA4 which also express the proliferation-associated stem cell markers- C-KIT and CD133 (Fig. 1D). In order to reveal the detailed methylation pattern of the 5′-regulatory region of the OCT4 gene in the UM51, we employed standard bisulfite sequencing. In total 330 Cytosine-phosphatidyl-Guanine-dinucleotides (CpG) upstream of the transcription-starting site (TSS) of the OCT4 gene were analysed. Within this 469 bp long region, a dense methylation pattern was observed in the UM51 cells, with 92.4% (305) of the CpG dinucleotides identified were methylated (Fig. 1E). In contrast, iPSCs derived from UM51 had 72.12% (207) of analysed CpGs were unmethylated (Supplemental Fig. S1B).

Urine-derived renal progenitors express the mesenchymal marker- Vimentin and not the epithelial marker- E-Cadherin (Fig. 1D, Supplemental Fig. S1C). Flow cytometry analysis of critical MSC cell surface markers were negative for the hematopoietic markers CD14, CD20, CD34, and CD45 and positive for CD73, CD90 and CD105 albeit at variable levels (Supplemental Fig. S1D). Typical of MSCs, urine-derived renal progenitor cells can also differentiate into osteocytes, chondrocytes, and adipocytes when cultured in the respective differentiation medium for 3 weeks (Fig. 1F, Supplemental Fig. S1E). Furthermore, employing a cytokine array (n = 2), a plethora of trophic factors such as IL8, GDF-15, SERPINE-1, Angiogenin, VEGF, and Thrombospondin-1 were detected, and further analysis of their associated GOs and KEGG pathways revealed immune system related terms (Fig. 1G, Supplemental Fig. S1F-G).

### Urine-derived renal progenitors express key renal progenitor cell markers and are able to endocytose Albumin

**Figure 2:**
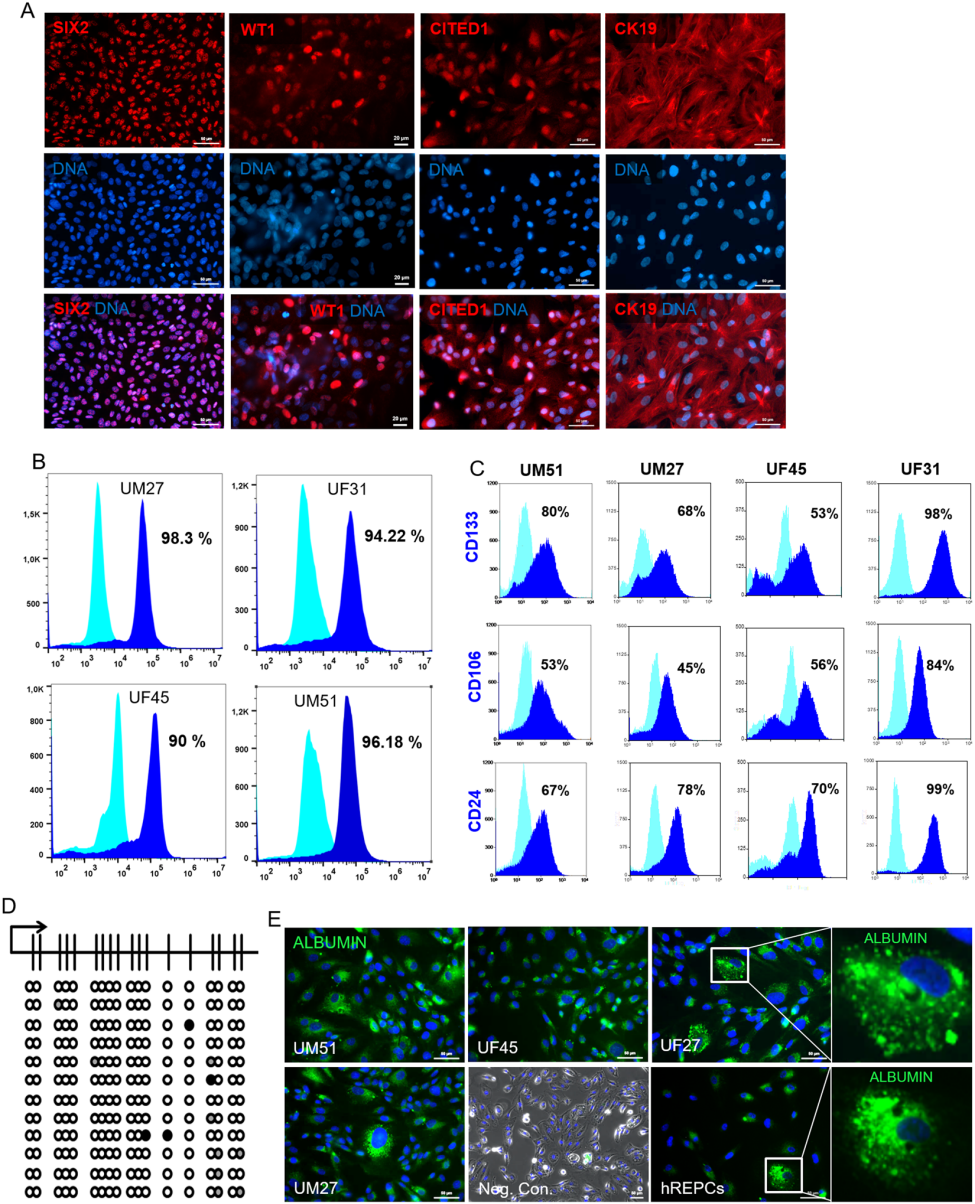
Expression of kidney-associated proteins in urine-derived renal progenitors and Albumin transport. **(A)** Urine-derived renal progenitors express the renal stem cell markers- SIX2, CITED1, WT1, and CK19. Renal markers (red) and cell nuclei were stained using DAPI/Hoechst (blue). **(B)** Flow cytometry analysis for the key renal stem cell transcription factor SIX2 and **(C)** Renal stem cell surface markers CD24, CD133, and CD106 of UM27, UF31, UF45 and UM51. **(D)** Detailed CpG methylation profiles of the SIX2 5′-regulatory region are documented as revealed by bisulfite sequencing. Filled circles represent methylated CpG dinucleotides and white circles unmethylated CpGs. Arrows indicate the transcription start site. 1.9% of CpG dinucleotides were found to be methylated. **(E)** Urine-derived renal progenitors (n = 4) like the human kidney biopsy-derived hREPCs also transport Albumin. Albumin was coupled to Alexa Fluor 488 (green) and cell nuclei stained with DAPI (blue). Scale bars indicate 50 µm.

Immunofluorescence-based staining revealed expression of the key renal stem cell proteins such as CK19 and the transcription factors- SIX2, CITED1, WT1, as shown by representative images (Fig. 2A). To determine the variability of SIX2^+^cells between the progenitor cell preparations- UM27, UF31, UM51 and UF45 (n = 4) a flow cytometry analysis was performed. We observed approximately 95% SIX2^+^ cells in UM27, UF31 and UM51 whereas UF45 had 90% SIX2^+^ cells (Fig. 2B). In addition, to confirm the renal stemness status of the urine-derived progenitor cells a flow cytometry analysis was performed to evaluate expression of the renal progenitor markers CD24, CD106 and the self-renewal marker CD133 in the cell preparations- UM27, UF31, UM51 and UF45. CD24, CD133 and CD106 were variably expressed in the aforementioned cell preparations. For instance, 98% of the UF31 cell population was CD133^+^, 99% of the cells were positive for CD24 and 84% of the cells were CD106^+^. On the other hand, the UF45 sample displayed a different pattern for CD133 (68%), CD24 (70%) and CD106 (45%) positive cells, respectively (Fig. 2C). Bisulfite sequencing of a portion of the 5′-regulatory region of the SIX2 gene revealed methylation of only 1.9% of CpG dinucleotides (Fig. 2D). As, presence of albumin in urine is a mark of kidney cell functionality, and by the endo/exocytosis of albumin, kidney maintain the colloid osmotic pressure and transport biomolecules. We performed endocytosis assay and could show that urine-derived cells can transport Albumin (Fig. 2E). Furthermore, the CYP2D6 genotypes investigated were distinct between groups of individuals, thus reflecting potential diverse drug metabolizing activities. UM51 for example expresses the CYP2D6 *4/*17 genotype which confers an intermediate metabolizing activity whereas UF31 bears the CYP2D6*1/*41 genotype with an ultra-rapid metabolizing activity. The other three individuals (UF21, UF45 and UM27) are endowed with normal drug metabolizing activity (Supplemental Table S1).

### Comparative transcriptome analysis of urine-derived renal progenitors and kidney-biopsy derived renal epithelial proximal cells (hREPCs)

**Figure 3:**
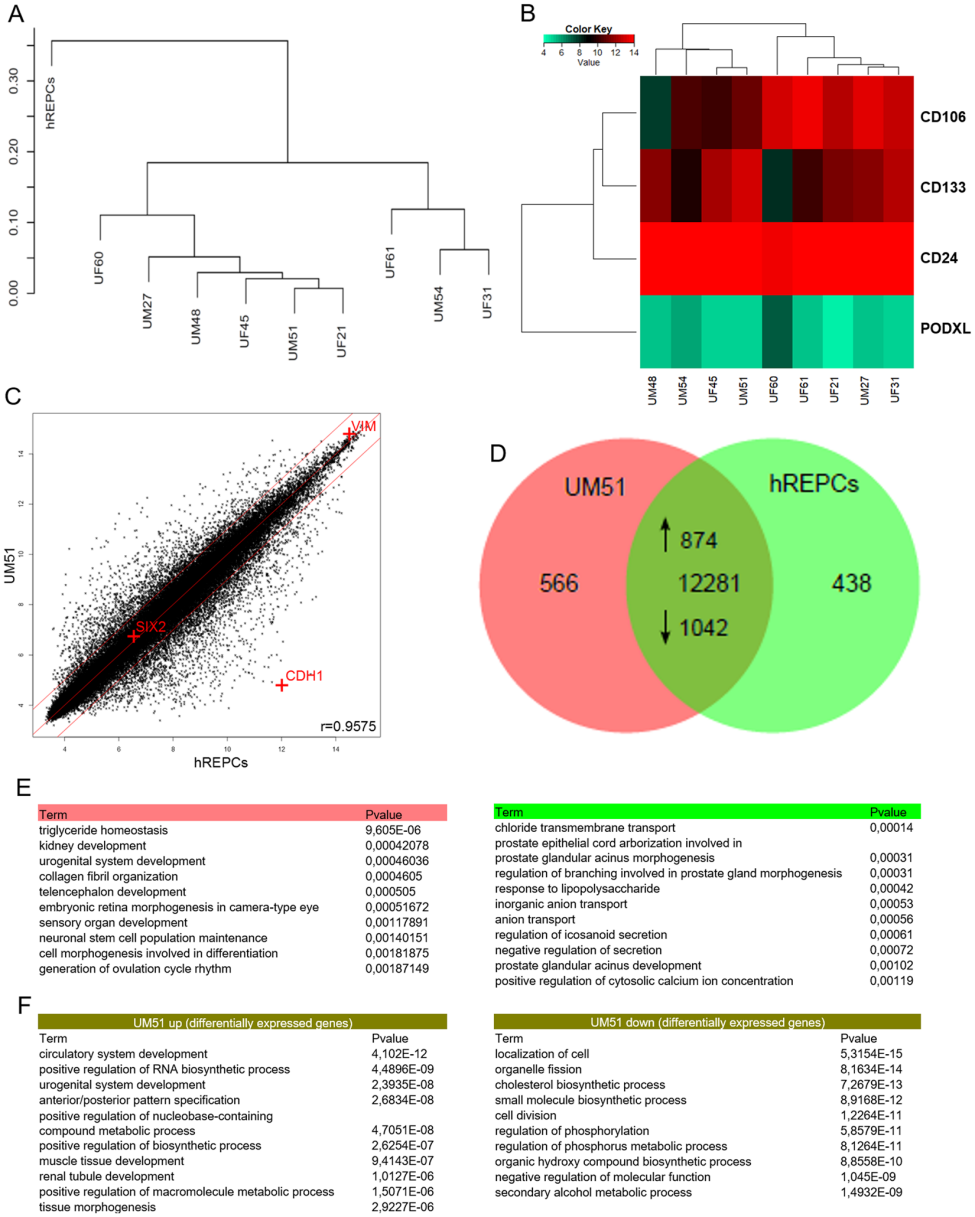
Transcriptome analysis of urine-derived renal progenitors in comparison to kidney biopsy-derived renal epithelial proximal cells- hREPCs. **(A)** A hierarchical cluster dendrogram based on transcriptomes of urine-derived renal progenitors with the kidney biopsy-derived renal epithelial proximal cells (hREPCs). **(B)** The heatmap of renal progenitor cell surface markers (*CD24, CD133, and CD106*) expressed in urine-derived renal progenitors. **(C)** Comparison of gene expression values of urine-derived renal progenitors (UM51) with hREPCs in a scatter plot confirms the mesenchymal phenotype of urine-derived renal progenitors, i.e. expression of Vimentin (*VIM*) and expression of E-cadherin (*CDH1*) in hREPCs. **(D)** Expressed genes (det-p < 0.05) in urine-derived renal progenitors (sample UM51) and hREPCs are compared in the Venn diagram. **(E)** The 10 most over-represented GO BP-terms in 566 UM51 genes include triglyceride homeostasis and kidney development and in 438 hREPCs genes include chloride transmembrane transport. **(F)** The 10 most over-represented GO BP-terms in the up- and down-regulated genes in UM51 in comparison to hREPCs are shown. The complete dataset is presented in Supplemental Table S4.

A hierarchical clustering analysis comparing the transcriptomes of urine derived renal progenitors with the kidney biopsy-derived renal epithelial proximal cells (hREPCs) revealed that all urine derived renal progenitors samples clustered together as a common cell type distinct from hREPCs (Fig. 3A). Additionally, expression of renal progenitor surface markers *CD24*, *CD106* and *CD133* were detected in urine-derived renal progenitors whilst *PODXL* was not expressed (Fig. 3B). These renal progenitors are of mesenchymal origin expressing *VIM*, however a scatter plot comparison between UM51 with hREPCs shows similarity with a high Pearson correlation of 0.9575 (Fig. 3C). The epithelial character of hREPCs is reflected by *CDH1* expression. The comparison of expressed genes (det-p < 0.05) in renal progenitors (UM51) and hREPCs in a venn diagram revealed a common 12281 gene-set, whereas 566 are expressed exclusively in UM51 and 438 exclusively in hREPCs (Fig. 3D). The 10 most over-represented GO BP terms (biological processes) in the UM51 exclusive gene-set include triglyceride homeostasis, kidney development and urogenital system development, whereas the hREPCs exclusive gene set includes chloride transmembrane transport, anion transport and response to lipopolysaccharides (Fig. 3E). The common gene set consists of 874 up-regulated genes (ratio > 2) in UM51 (e.g. renal tubule development, urogenital system development and anterior/posterior pattern specification) and 1042 down-regulated genes (ratio < 0.5) in UM51 (e.g. cell division and cholesterol biosynthetic process) (Fig. 3F).

### Comparative gene expression analysis of urine-derived and kidney biopsies-derived renal progenitor cells

Gene expression of urine-derived renal progenitors was compared to public available datasets GSE23911 in which nephron progenitor cells were derived from adult human renal cortical tissue^52^. Additionally, the comparison was extended by two further datasets GSE74450 and GSE75949 which contain data from fetal kidney biopsy derived nephron progenitor cells^53,54^. We could show that urine-derived renal progenitors have a high level of similarity to other human nephron progenitors at the transcriptome level. The resulting number of expressed genes were comparable: 12112 genes in urine-derived renal progenitors, 8446 genes in GSE23911, 10597 genes in GSE74450 and 13895 genes in GSE75949. In the Venn diagram analysis most genes were found in the intersection of all genesets (4411), followed by the intersection of urine-derived renal progenitors with the fetal kidney genesets from GSE74450 and GSE75949. Among the intersection with single genesets urine-derived renal progenitors had the highest overlap with the GSE75949 pointing at the highest similarity with this geneset (Supplemental Fig. S2). A subset of genes expressed in common between urine-derived renal progenitors, GSE74450 and GSE75949, are associated with renal system development related GO’s (BP) terms, thus confirming renal progenitor cell identity (Supplemental Table S5).

### Confirmation of the renal origin of urine-derived progenitor cells and retention of renal-associated genes in urine-derived progenitors-iPSCs

**Figure 4:**
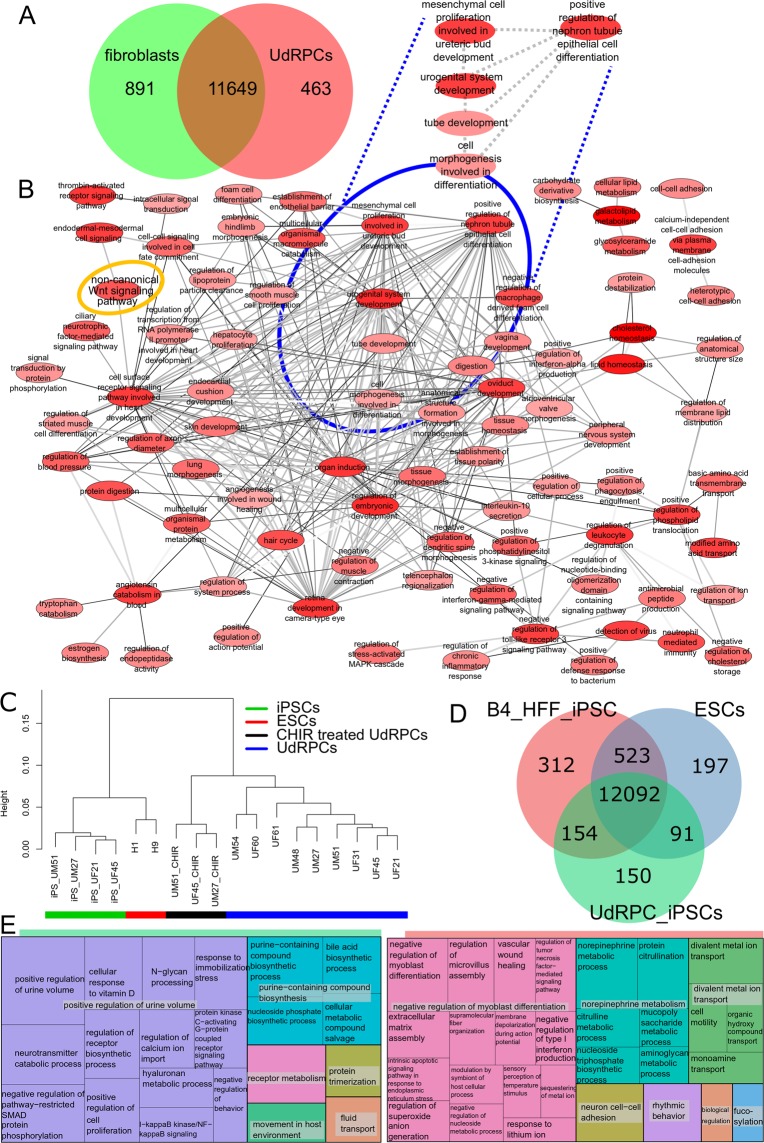
In-depth bioinformatic analysis of urine-derived renal progenitors and urine-derived renal progenitors derived-iPSCs. **(A)** Expressed genes (det-p < 0.05) in urine-derived renal progenitors (UdRPCs) and fibroblasts are compared in a venn diagram. Most genes are expressed in common (11649), 463 genes are expressed exclusively in urine-derived renal progenitors and 891 in fibroblasts. The subsets and urine-derived renal progenitors GOs are presented in supplemental_table_S4. **(B)** The gene ontology network was generated with the tools REVIGO and Cytoscape and summarizes the GO terms of category Biological Process (BP) over-represented in the 463 genes expressed exclusively in urine-derived renal progenitors. Several general developmental terms emerged, e.g. “organ induction”. Specific renal-related terms including “urogenital system development” are marked with a blue ellipse. GOs are represented by the network nodes with the intensity of red indicating the significance of over-representation of a GO term. The edges refer to similarities between the GO terms. **(C)** The dendrogram shows a clear separation of urine-derived renal progenitors, differentiated urine-derived renal progenitors (CHIR treated UdRPCs of UM51, UM27, and UF45) (black bar), ESCs (H1 and H9, red bar) and urine-derived renal progenitors-iPSCs (green bar). **(D)** Venn diagram of HFF-iPSCs, urine-derived renal progenitors-iPSCs (UdRPCs_iPScs) and ESCs. **(E)** GO terms of 150 genes expressed exclusively in urine-derived renal progenitors-iPSCs indicate that these iPSCs retain the memory of renal origin. In the treemap for the HFF-iPSCs the GO-BP terms of the 312 over-represented genes of the exclusive gene set are summarized. The most significant group is associated with negative regulation of myoblast differentiation including genes *DDIT3*, *MBNL3*, *TGFB1*, *ZFHX3* pointing at the fibroblast origin of these iPSCs. The entire dataset is presented in Supplemental Table S7.

A venn diagram-based comparison of gene expression (det-p < 0.05) in urine-derived renal progenitors and human foreskin fibroblasts (HFF) was carried out (Fig. 4A) in order to dissect common and distinct gene expression patterns. The majority of genes (11649) are expressed in common, 463 exclusively in urine-derived renal progenitors and 891 in fibroblasts. The 463 genes were further analysed for over-represented GOs and summarized as a GO network (Fig. 4B) with the tools REVIGO, and Cytoscape was used for the GO terms of the category BP. In addition to several developmental terms such as organ induction, regulation of embryonic development (high number of edges referring to similarity to many terms), specific renal-related terms including urogenital system development, mesenchymal cell proliferation involved in ureteric bud development and positive regulation of nephron tubule epithelial cell differentiation (marked with blue ellipse, intense red indicating higher significance) were identified. Interestingly, the non-canonical WNT signalling pathway, which plays a major role in kidney development, is also over-represented (orange ring-top left).

The dendrogram based on the global transcriptome analysis revealed a clear separation of urine-derived renal progenitors lines (n = 9) from the differentiated urine-derived renal progenitors (CHIR 99021 treated urine- derived renal progenitor cells, n = 3), urine derived renal progenitors-iPSCs (n = 4) and embryonic stem cells (H1 and H9) (Fig. 4C). Characterization of the derived urine derived renal progenitors-iPSCs is depicted in Supplemental Fig. S3. In the Venn diagram (Fig. 4D) we compared expressed genes (det-p < 0.05) in urine derived renal progenitors-iPSCs with ESCs and HFF-iPSCs. Most genes (12092) are expressed in common in all cell types while 150 genes are expressed exclusively in urine derived renal progenitors-iPSCs. The genes expressed exclusively in one cell type were further analysed for over-representation of GO terms. The treemap summarizing the GO terms of category BP over-represented in the 150 genes expressed exclusively in urine derived renal progenitors-iPSCs (Fig. 4E) indicates that these iPSCs retain a memory of their kidney origin. In addition to the largest most significant group- positive regulation of urine volume, it consists of other renal-related GO terms (e.g. calcium transport, vitamin D). Stem-cell-related and developmental terms such as positive regulation of cell proliferation are due to their pluripotent nature. Within the treemap summarizing the GO-BP terms over-represented in the 312 genes expressed exclusively in HFF-iPSCs, the largest most significant group is associated with negative regulation of myoblast differentiation, thus pointing at the fibroblast origin of these iPSCs (Fig. 4E). Furthermore, within the treemap summarizing the GO-BP terms over-represented in the 197 genes expressed exclusively in ESCs, the largest most significant group is associated with negative regulation of astrocyte differentiation- hinting at their known propensity to differentiate into the ectodermal lineage (Supplemental Fig. S4).

### WNT pathway activation by GSK3β inhibition induces differentiation of urine-derived renal progenitors into renal epithelial proximal tubular cells

**Figure 5:**
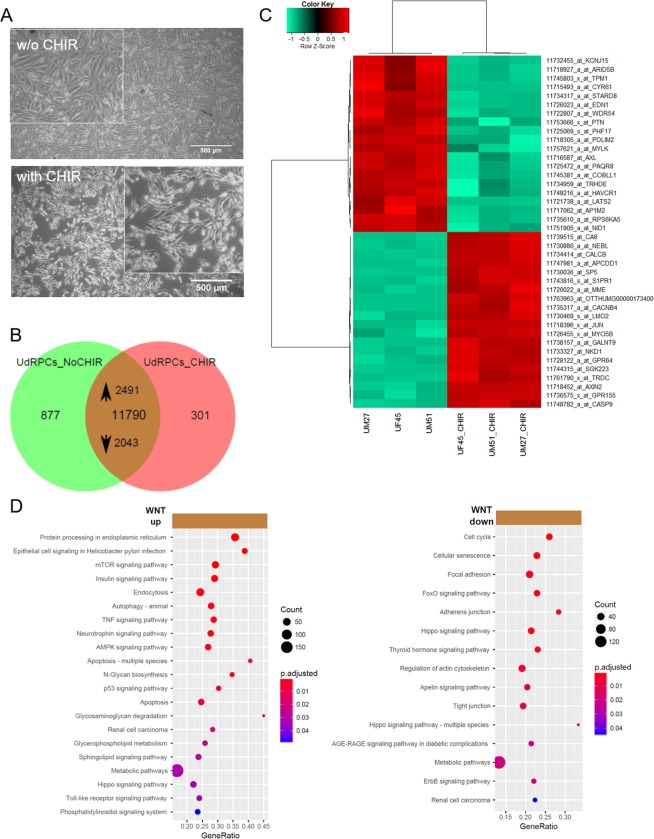
Supplementation of urine-derived renal progenitors with the GSK-3β inhibitor. **(A)** Activation of WNT signalling by supplementation with GSK-3β-inhibitor CHIR99021 led to differentiation into renal epithelial proximal tubular cells. **(B)** In the Venn diagram, expressed genes (det-p < 0.05) in untreated urine-derived renal progenitors (UdRPCs_NoCHIR) are compared to urine-derived renal progenitors treated with the GSK-3β-inhibitor CHIR99021 (UdRPCs_CHIR). Among the 11790 genes expressed in both conditions, 2491 are up-regulated in the CHIR99021 treatment (p < 0.05, ratio > 1.33) and 2043 down-regulated (p < 0.05, ratio < 0.75). **(C)** Heatmap of 3 independent urine-derived renal progenitor cell preparations with and without CHIR treatment. **(D)** Over-representation analysis of the up-regulated genes and associated KEGG pathways revealed protein processing in endoplasmic reticulum as highly significant and several signalling and metabolic pathways including mTOR, Insulin, p53 and TNF. Over-representation analysis of the down-regulated genes in KEGG pathways identified cell cycle, cellular senescence, focal adhesion, FoxO and adherens junction as most significant. Supplemental Table S8 provides the full list of regulated genes and associated pathways.

To differentiate three independent renal progenitors preparations, the cells were treated with 10 μM CHIR99021 (WNT pathway activation by GSK3β inhibition) for 2 days and morphological changes from fibroblastic to elongated tubular shape were observed (Fig. 5A). In the Venn diagram, expressed genes (det-p < 0.05) in untreated urine-derived renal progenitors are compared to renal progenitors treated with CHIR99021. Genes expressed in common amounts to 11790, of these 2491 are upregulated in the CHIR99021 treatment (p < 0.05, ratio > 1.33) and 2043 are down-regulated (p < 0.05, ratio < 0.75) (Fig. 5B, Supplemental Table S8). Among the upregulated genes, 27 are considered “novel” (gene symbol starting with “LOC”), 21 among the down-regulated genes and 98 among the non-regulated genes (Supplemental Table S8). The heatmap based on the top 20 regulated genes shows a clear separation between untreated and treated cells (Fig. 5C). Amongst the up-regulated genes, the associated KEGG pathways include WNT-signaling (*AXIN2*, *JUN*, *NKD1*) (Supplemental Fig. S5). Over-representation analysis of the up-regulated genes and their associated KEGG pathways identified protein processing in endoplasmic reticulum as highly significant and several signalling pathways such as mTOR, Insulin, p53, AMPK and TNF. Over-representation analysis of the down-regulated genes and associated KEGG pathways revealed cell cycle, cellular senescence, focal adhesion, FoxO, ErbB and thyroid hormone signalling. Interestingly Hippo pathway was regulated in both undifferentiated and differentiated renal cells (Fig. 5D).

### Regulation of self-renewal and differentiation in urine-derived renal progenitor cells

**Figure 6:**
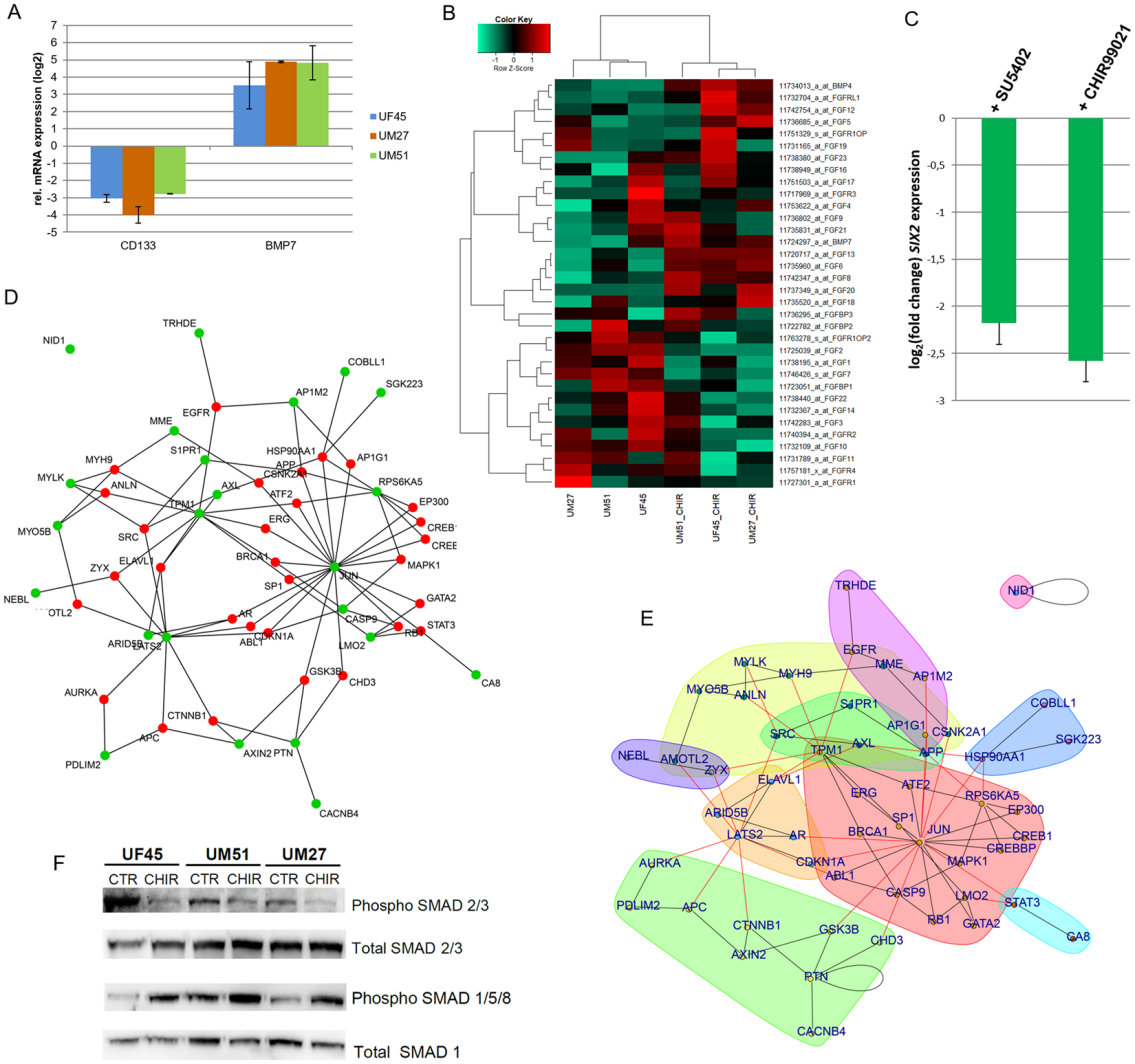
Regulation of self-renewal and differentiation in urine-derived renal progenitors. **(A)** Real-time PCR-based confirmation of down-regulation of *CD133* and activated expression of *BMP7* after CHIR stimulation. **(B)** Effect of CHIR99021 stimulation on FGF-signaling and BMP (BMP7 and BMP4) signaling. The heatmap depicts FGF signaling associated genes up and down regulated upon CHIR treatment of the urine-derived renal progenitors. **(C)** Downregulation of *SIX2* expression in differentiated urine-derived renal progenitors upon WNT stimulation using the GSK-3β-inhibitor CHIR99021 and blocking of FGF signaling using the FGF receptor inhibitor SU5402. **(D)** JUN is a major hub of protein interaction networks of urine-derived renal progenitors treated with CHIR. Based on the Biogrid database protein interaction networks were constructed from the set of the most highly regulated 40 genes either up- or down in the urine-derived renal progenitors treated with CHIR. The selected genes used to connect to the network with interactions from the Biogrid database are marked in green, genes added as Biogrid interactions are marked in red. Induction of WNT leading to GSK3B inhibition is reflected by the connection of GSK3B to JUN and to AXIN2 which is connected to CTNNB1 (β-catenin) – these all downstream targets of GSK3B in the WNT-signaling pathway. **(E)** Community clustering of the network identified several communities: JUN (red), GSK3B/AXIN2/CTNNB1 (green), LATS2 (yellow), EGFR (pink). Black lines refer to edges within a community, red lines to edges between different communities. **(F)** Western blot analysis of the phosphorylated levels of SMAD 2/3 and SMAD 1/5/8 in undifferentiated and differentiated UF45, UM51 and UM27.

Further to the transcriptome analyses, a real-time PCR revealed downregulation of the stem cell self-renewal associated gene *CD133* and activated expression of the nephrogenesis-associated gene *BMP7* after CHIR99021 stimulation (Fig. 6A). Since FGF signaling is also crucial for maintaining self-renewal, we compared the transcriptome of differentiated cells (CHIR99021 treated) and progenitor cells to investigate the effect of CHIR99021 stimulation on FGF-signaling with respect to the genes from FGF and FGFR family; BMP7 and BMP4 from the BMP family. We detected an upregulation of FGF2 and FGF7 in undifferentiated renal progenitors (Fig. 6B). To validate this, we disrupted FGF signaling using fibroblast growth factor receptor (FGFR) inhibitor SU5402, and observed morphological changes (Supplemental Fig. S6). Interestingly, downregulation of the key renal transcription factor *SIX2* was detected in both the CHIR99021 and SU5402 treated cells (Fig. 6C). Furthermore, to identify the self-renewal regulators and pathways in urine-derived renal progenitor cells, a protein-protein-interaction network was generated. The network of the 40 proteins, encoded by the 20 most significantly up- and down-regulated genes between CHIR99021 treated and untreated urine-derived renal progeitors (Fig. 5C) indentified JUN as a major hub – in terms of having most connections to other proteins in the network. However, in the WNT-signaling pathway JUN is at the end of a downstream cascade from GSK3β, including further downstream targets- AXIN2 and CTNNB1. The genes encoding these proteins were differentially regulated by the CHIR99021 treatment (green nodes) (Fig. 6D). Several communities with more interactions within the community than to other communities can be detected in the network via community clustering of the network via edge-betweenness includes JUN (red), GSK3β / AXIN2 / CTNNB1 (green), LATS2 (yellow), EGFR (pink) (Fig. 6E). To analyze the effect of WNT activation on the TGFβ-SMAD pathway, Western blot analysis was performed to detect phosphorylation levels of SMAD 2/3 and SMAD 1/5/8 in UF45, UM51 and UM27. In the differentiated cells (urine-derived renal progenitors after CHIR treatment) a decreased level of phosphorylated SMAD 2/3 and increased levels of phosphorylated SMAD 1/5/8 were observed (Fig. 6F).

## Discussion

Although relatively few renal cells are shed under healthy conditions compared to dysfunctional conditions^10,55^, we were able to isolate, culture and expand urine cells from healthy donors. Here we describe urine as a reliable, non-invasive, robust and cheap source of renal stem cells, in contrast to amniotic fluid or kidney biopsies^56,57^. Urine derived stem cells can be expand from a single clone with high proliferation potency^37,58^. We propose naming these cells as urine-derived renal progenitor cells, because they can be kept in culture for almost 12 passages whilst maintaining expression of the self-renewal associated proteins- SIX2, CITED1, CD133, C-KIT, TRA-1-60, TRA-1-81 and SSEA4 as has been shown by others^37,56^. Despite the expression of a subset of pluripotency-associated factors, these renal progenitor cells do not express OCT4, SOX2 and NANOG- which are key pluripotency-regulating transcription factors^59,60^. Further evidence in support of the lack of OCT4 expression is our observed fully methylated CpG dinucleotides within the OCT4 promoter in the UM51 cells. Urine-derived renal progenitors are in fact bon-fide MSCs- i.e. they express VIM and not CDH1, adhere to plastic surfaces, express CD73, CD90 and CD105 and not the hematopoietic markers CD14, CD20, CD34, and CD45. Typical of MSCs, urine-derived renal progenitors can be differentiated into osteoblasts, chondrocytes and adipocytes^56,57,61^. They also secrete a plethora of cytokines and growth factors- such as EGF, GDF, PDGF and Serpin E1^62^. The multipotent features of urine-derived renal progenitors make these cells promising for studying nephrogenesis and in the future regenerative therapy of kidney-associated diseases.

Urine-derived renal progenitor cells express key renal progenitor-regulatory proteins SIX2, CITED1 and WT1 indicating they originate from the kidney as described from others^13,14,27,57,63^. Unmethylated CpG islands within the 5′- regulatory region of the SIX2 gene confirm their progenitor status. Nuclear-localized SIX2 expression is critical for maintaining self-renewal of renal stem cell populations and has been described to co-localize with CITED1^25^. We observed CITED1 expression in both the nucleus and cytoplasm, this is supported by a subcellular fractionation study that demonstrated an abundant portion of CITED1 localized in the cytoplasm whereas only 5% were expressed in the nucleus^64^. As CITED1 is a cell cycle-dependent transcriptional co-factor and contains a nuclear export signal domain, the subcellular localization might be dependent on its phosphorylation status. We previously, showed CITED1 and WT1 expression in the nucleus and cytoplasm of amniotic fluid cells of renal origin^56^. Here, we observed that subpopulations of adult urine-derived progenitor cells express WT1 in the nucleus and in some cases, both nuclear and cytoplasmic localization was observed. Wt1 has nucleocytoplasmic shuttling activity, however, the shuttling of Wt1 between the nucleus and cytoplasm might regulate the activity as a transcription factor as a result of interaction with the cargo protein importins α1 and β^65,66^.

In line with our study, urine-derived renal progenitor cells have been described to express the surface marker CD24, CD106, and CD133^16^. However, we observed variabilities in the numbers of cells expressing these markers between preparations. This variance might be due to the origin of the urine- shed cells in the adult^4,6,7,67^. For example, CD24 and CD133 positive cells have been found in renal tubules and the renal capsule, but CD106^+^ cells are only present in the renal capsule.

Furthermore, urine-derived renal progenitors transport albumin^56,68^. The albumin filtration pathway partly takes place in the kidney and the presence of albumin in urine is used as a marker for cell functionality as described by the endo/exocytosis of albumin in kidney^69^. The GOs derived from the exclusively expressed genes in urine-derived renal progenitors (compared to HFF1) unveiled renal system development- related terms. To overcome the lack of reference human kidney biopsy-derived renal progenitors, we performed a meta-analysis comparing our data to nephron progenitor cell transcriptome datasets downloaded from NCBI GEO. The analyses revealed that our urine-derived renal progenitors share a high level of similarity with other human nephron progenitors at the transcriptome level^52–54^. Moreover, the GOs from the urine cell derived-iPSC exclusive genes-set, in contrast to pluripotent stem cells, identified terms related to renal function therefore implying the preservation of their kidney origin. As the conservation of tissue of origin in iPSCs might be linked to epigenetic memory^17,70^, urine-derived renal progenitors as well as corresponding-iPSCs, especially with known CYP2D6 status, might be advantageous for differentiation into renal cells, modelling kidney-related diseases, nephrotoxicity studies and regenerative medicine^55^.

Dissecting the gene regulatory mechanisms that drive human renal progenitor growth and differentiation *in vitro* represents the key step for translation but remains a challenge due to the absence of well-characterised primary urine derived stem cells. Here we have shown that urine-derived renal cells are a self-renewing stem cell population unlike the kidney biopsy-derived hREPCs which are differentiated renal epithelial cells. To demonstrate that urine-derived renal progenitors can maintain self-renewal when cultured under undifferentiation conditions but yet retain the potential for epithelial differentiation and nephrogenesis, we induced active WNT signalling, by treatment with the GSK-3β inhibitor- CHIR99021. The differentiated cells adopted an elongated tubular morphology and reduced proliferation as also shown for human ESC and iPSC derived renal epithelial cells^71–73^. Although WNT pathway activation induced an epithelial phenotype, we did not see a dramatic increase in *CDH1* expression at the time point and dose used but rather activation of *CDH-3* expression (8.86 fold). *Cdh-3*, a gene encoding a member of the cadherin superfamily, functions in epithelial cell morphogenesis in *Caenorhabditis elegans*^74^ an event which is poorly understood in human nephrogenesis. Furthermore, the correlation co-efficient (0.941) of the WNT-induced differentiated UM51 with hREPCs is further evidence in support of the cellular identify of the UM51 differentiated cells.

In line with our previously published observations in amniotic fluidic-derived renal cells, the down-regulated expression of *SIX2*, *WT1*, *CD133* and upregulated expression of *BMP7* induced the loss of self-renewal^56^. Global transcriptome analyses also revealed the down-regulation of 2043 genes some of which are associated with pathways such as cell cycle, FoxO, Hippo and ErbB signalling. The Hippo pathway which is composed of WNT target genes such as *LATS2, AXIN2* and *CTNNB1* have been reported to regulate epithelialization of nephron progenitors^75,76^.

We detected differential expression 40 genes in which 20 most significantly up- and down-regulated between WNT-induced differentiated and self-renewing urine-derived renal progenitors. Amongst the genes up regulated in the CHIR99021 treated cells are the WNT targets- *AXIN2*, *JUN* and *NKD1* known to be associated with WNT signalling. Interestingly, a protein interaction network identified JUN as a major hub connected to GSK3β and interlinked with ATF2, STAT3, GATA2 and MAPK1. In a mouse model, it has been reported that Bmp7 phosphorylates Jun and Atf2 via Jnk signalling which promote the proliferation of mouse nephron progenitors^33^. This indeed might be contradictory to our observed elevated expression of *BMP7* upon WNT induced differentiation of urine-derived renal progenitor cells- i.e. suppression of *BMP7* expression is needed to maintain self-renewal in urine-derived renal progenitor cells. However, in line with our results, during *in vitro* differentiation (mesenchymal to epithelial transition) of human renal cell line TK173, BMP7 is required for the activation of E-Cadherin and WNT4 expression^77^. Since, SMADs are a target of MAPK particularly of JNK, both BMPs and TGFβ can activate the SMAD circuit^78,79^. Both activation of the WNT pathway and inhibition of FGF signalling led to the down-regulation of the key renal progenitor self-renewal associated transcription factor SIX2 and the up-regulated expression of BMP7. This is in line with the reported interactions of BMP and FGF signalling during nephrogenesis^80–82^. Fibroblast growth factor signaling is essential for *in vivo* renal development as well as *in vitro* cultivation and maintenance of nephron progenitor cells as demonstrated by mouse model experiments where blocking of FGF receptors led to aberrant nephrogenesis^25,81^.

**Figure 7:**
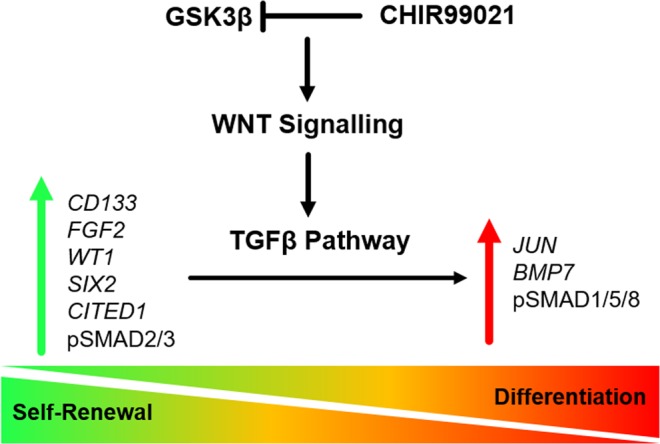
WNT/β-catenin and TGFβ pathway-mediated cell fate decisions in urine-derived renal progenitors. Self-renewal (inactive WNT/β-catenin signalling and active TGF-β/SMAD2/3 signalling) is maintained by elevated expression of the renal progenitor markers *SIX2*, *WT1*, *CITED1*, *CD133*, in addition to phospho-SMAD2/3 and FGF2 resulting in and down regulated expression of *BMP7*. In contrast, activation of WNT/β-catenin signalling induces upregulated expression of JUN and *BMP7* leading to activation of phospho-SMAD1/5/8, downregulated expression of *WT1, SIX2, CITED1, FGF2, CD133* and ultimately exit of self-renewal.

Based on the present study and our previously published data in human amniotic fluid-derived renal cells^56^, we propose that similar to self-renewal in human pluripotent stem cells^60,83^, urine-derived renal progenitor cells maintain self-renewal by active FGF signalling leading to phosphorylated TGFβ- SMAD2/3. In contrast, activation of WNT/β-catenin signalling leads to an upregulation of *JUN* and *BMP7* leading to activation of SMAD1/5/8 signalling and exit of self-renewal by downregulation of *WT1, SIX2*, *CITED1*, and *CD133* expression. To surmise, we derived a hypothetic scheme of the WNTβ catenin and TGFβ pathway-mediated cell fate decisions in urine-derived renal progenitor cells. This simplistic model is depicted in Fig. 7.

Comparing self-renewal of renal progenitor cells in both human (urine-derived renal progenitors) and mouse, it is clear that an intricate balance is needed between SIX2, WT1, CITED1 expression and Wnt/β-catenin activity in order to determine the cell fate of nephron progenitor cells^24,31,34,56^. Furthermore, it remains to be determined if indeed there exist subtle human and mouse differences in the gene regulatory network needed to maintain a self-renewing renal progenitor pool in both species and we believe that human urine-derived renal progenitor cells as described here will facilitate these studies.

## Materials and methods

### Ethics statement

In this study, urine samples were collected with the informed consent of the donors and the written approval (Ethical approval Number: 5704) of the ethical review board of the medical faculty of Heinrich Heine University, Düsseldorf, Germany. All methods were carried out in accordance with the approved guidelines. Medical faculty of Heinrich Heine University approved all experimental protocols.

### Isolation, culture, and differentiation of urine-derived renal progenitor cells

Urine samples were collected from 10 healthy donors with diverse age, gender and ethnicity (Supplemental Table S1). Isolation and expansion of the urine-derived renal progenitors followed the previously established protocols^37,41^. For differentiation of the urine derived renal progenitors, 10 µM CHIR99021 was added to the cell culture medium for 2 days. Adult kidney biopsy derived primary human renal epithelial cells (hREPCs) (C-12665, Promo Cell, Germany) were used as control. To inhibit FGF signaling in urine derived renal progenitors, 15 µM SU5402 was added to the cell culture medium for 2 days.

### Immunofluorescence staining

Immunofluorescence study was performed as described previously^84^. To analyse expression of specific markers, at 80% confluence, attached urine-derived renal progenitor cells of four individuals (Passages 4-5) were fixed with 4% PFA (Polysciences Inc., USA) for 15 min at room temperature (RT) and washed three times in PBS and permeability was increased using 1% Triton X-100 for 5 min. Next, for blocking we used: 10% normal goat serum (NGS; Sigma-Aldrich Chemie GmbH, Germany), 0.5% Triton X-100, 1% BSA (Sigma-Aldrich Chemie GmbH, Germany) and 0.05% Tween 20 (Sigma-Aldrich Chemie GmbH, Germany) in PBS for 2 h. The cells were incubated with primary antibodies (Supplemental Table S2) for 1 h at RT followed by three washes with PBS. Thereafter, the corresponding secondary Cy3-labeled, Alexa Fluor-555 or Alexa Fluor 488-labeled antibodies (Thermo Fisher Scientific, USA) and Hoechst 33,258 dye (Sigma-Aldrich Chemie GmbH, Germany) or DAPI (Southern Biotech, USA) were added. A fluorescence microscope (LSM700; Carl Zeiss Microscopy GmbH, Germany) was used for taking the pictures. All pictures were processed with the ZenBlue 2012 Software Version 1.1.2.0. (Carl Zeiss Microscopy GmbH, Germany).

### CYP2D6 genotyping/phenotyping and Albumin endocytosis assay

CYP2D6 genotyping and phenotyping of five individuals (randomly selected) were carried out by CeGat GmbH Germany using genomic DNA. The CYP2D6 variant assay reveals the pharmacogenetics (PGx) profile of an individual’s genotype and phenotype based on tested pharmacogenetics markers. The assay identifies and discriminates individuals with poor, normal, intermediate and ultra-rapid metabolizing activity^85^. Albumin endocytosis assay was performed as described before^56^. For detailed description, see supplemental materials and methods.

### Immunophenotyping by flow cytometry

At 90% confluence, adherent cells at passage 3–5 from UM27, UF31, UF45 and UM51 were detached from 6-well plates by incubation in TrypLE (Thermo Fisher Scientific, USA) at 37 °C. Then the cell samples were subjected to fluorescence-activated cell sorting (FACS) in order to specifically select for MSC cell surface markers, renal stem cell transcription factor SIX2^+^ cells, and renal stem cell surface markers. Unstained cells and IgG isotype served as control for each cell sample. Dead cells and debris were gated on a two physical parameter dot plot followed by the exclusion of doublets by using pulse processing. Finally, the experimental cells positive subpopulation was gated. Sorting was done using CytoFLEX cell sorter (Beckman Coulter, USA), BD FACSCanto (BD Biosciences, Germany) and CyAn ADP (Beckman Coulter, USA). Histograms were generated using the Summit 4.3.02 software.

The analysis of MSC-associated cell surface marker expression of urine-derived renal progenitors was performed using MSC Phenotyping Kit (Miltenyi Biotec GmbH, Germany) according to the manufacturer’s instructions and as described before^84^. For the pluripotency-associated markers, TRA-1-60, TRA-1-81, and SSEA4 dye-coupled antibodies were used (anti-TRA-1-60-PE, human (clone: REA157), number 130-100-347; anti-TRA-1-81-PE, human (clone: REA246), number 130-101-410, and anti-SSEA-4-PE, human (clone: REA101), number 130-098-369; Miltenyi Biotec GmbH, Germany). For SIX2^+^ cell sorting, after blocking with Human TruStain blocking solution (Biolegend, USA) (5 µL each) for 10 min at RT, the cells (10^4^ cells/condition) were stained with anti-m-SIX2 (Abnova, Taiwan) primary antibody overnight at 4 °C. After 3 times washing with the Permwash buffer (Invitrogen, Germany), mouse Alexa-Fluor 488 was conjugated by incubating 30 min at RT in the dark. For the renal stem cell surface markers anti-CD24-FITC (Sigma-Aldrich Chemie GmbH, Germany), VCAM-1/CD106-PE (R&D systems, USA) and CD133-APC (R&D systems, USA) were used according to manufacturer instructions for flow cytometry analysis. Briefly, after blocking and washing, cells were centrifuged at 300 x g for 10 min. 5 µl of antibody solution (1:50 dilution) were added to the cell suspension and the samples were incubated in the dark at 4 °C for 10 min. Cells were washed afterwards, and stored in 4% PFA at 4 °C until analysis.

### Differentiation into adipocytes, chondrocytes and osteoblasts

Differentiation of urine-derived renal progenitors into adipocytes, chondrocytes and osteoblasts were tested using the StemPro Adipogenesis, Chondrogenesis, and Osteogenesis differentiation Kits (Gibco, Life Technologies, USA) as described before^56,84^. After the differentiation periods, cells were fixed using 4% PFA for 20 min at RT and stained with Oil Red-O for detecting adipocytes, Alcian Blue for chondrocytes, and Alizarin Red S for osteoblasts. A light microscope was used for imaging.

### Western blot analysis

For protein extraction, cells were harvested and lysed in RIPA buffer (Sigma-Aldrich Chemie GmbH, Germany) supplemented with complete protease and phosphatase inhibitors cocktail (Roche, Switzerland). The lysates were separated on a 4–20% Bis-Tris gel and blotted onto a 0.45 µm nitrocellulose membrane (GE Healthcare Life Sciences, Germany). The membranes were then blocked with 5% skimmed milk in Tris-Buffered Saline Tween (TBS-T) and incubated overnight with the respective primary antibodies: Total Smad 1 (1:1000, TBS-T 5% BSA; CST, USA), phospho Smad 1/5/8 (1:1000, TBS-T 5% milk; CST, USA), Total Smad 2/3 (CST, 1:1000, TBS-T 5% BSA), and phospho Smad 2/3 (1:1000, TBS-T 5% milk; CST, USA). After incubation with the appropriate secondary antibodies, signals were acquired with a Fusion-FX7 imaging system.

### Bisulfite genomic sequencing

Bisulfite sequencing was performed following bisulfite conversion with the EpiTec Kit (Qiagen, Germany). Primers were designed after excluding pseudogenes or other closely related genomic sequences which could interfere with specific amplification by amplicon and primer sequences comparison in BLAT sequence database (https://genome.ucsc.edu/FAQ/FAQblat.html). See supplemental materials and methods for full description.

### Generation of iPSCs from urine-derived renal progenitors

Four urine-derived renal progenitor cell samples were reprogrammed into iPSCs (four lines) using an integration-free episomal based transfection system without pathway inhibition. Briefly, urine-derived renal progenitor cells were nucleofected with two plasmids pEP4 E02S ET2K (Addgene plasmid #20927) and pEP4 E02S CK2M EN2L (Addgene plasmid #20924) expressing a combination of pluripotency factors including OCT4, SOX2, LIN28, c-MYC, KLF4, and NANOG using the Amaxa 4D-Nucleofector Kit (Lonza, Swiss) according to the manufacturer’s guidelines and as described previously^41^. Please see supplemental materials and methods for full description.

### Quantitative RT-PCR analysis

RNA was isolated using the Direct-zol RNA MiniPrep Kit (Zymo Research, USA) according to provider guidelines. After checking the quality of mRNA, 500 ng of RNA were used for complementary DNA synthesized with the TaqMan Reverse Transcription Kit (Applied Biosystems, USA). Real-time quantitative PCR was performed in technical triplicates with Power SYBR Green Master Mix (Life Technologies, USA), 12.5 ng cDNA per sample and 0.6 μM primers on a VIIA7 (Life Technologies, USA) machine. Mean values were normalized to levels of the housekeeping gene ribosomal protein L37A calculated by the 2−ΔΔCt method. Primers used were purchased from MWG (Supplementary Table S3).

### Microarray data analyses

Total RNA (1 μg) preparations were hybridized on the PrimeView Human Gene Expression Array (Affymetrix, Thermo Fisher Scientific, USA) at the core facility Biomedizinisches Forschungszentrum (BMFZ) of the Heinrich Heine University Düsseldorf. The raw data was imported into the R/Bioconductor environment^86^ and further processed with the package affy^87^ using background-correction, logarithmic (base 2) transformation and normalization with the Robust Multi-array Average (RMA) method. For full details, please see supplemental materials and methods.

### KEGG pathway, GO and network analysis

Gene ontology (GOs) terms were analysed within the Bioconductor environment employing the package GOstats^88^. GOs of category Biological Process (BP) were further summarized with the REVIGO tool^89^ to generate treemaps populating the parameter for allowed similarity with tiny = 0.4. GO networks were generated from the REVIGO tool in xgmml format and imported into Cytoscape^90^. For full details, see supplemental materials and methods.

### Activated WNT pathway associated protein interaction network

The network was constructed from the 20 most significantly up- and down down-regulated genes between CHIR99021 treatment and untreated controls. Genes were ranked by the limma-p-value and passed the criteria: detection p-value < 0.05 for the dedicated condition, ratio < 0.75 or ratio > 1.33, limma-p-value < 0.05. The resulting 40 genes were marked as green nodes in the network. Interacting proteins containing at least one protein coded by the 40 genes were retrieved from BioGrid version 3.4.161^91^. The plot of the interactions network was drawn employing the R package network^92^. See supplemental materials and methods for full description.

### Additional materials and methods

For the materials and methods of the culture supernatant analysis, analysis of cell proliferation, meta-analysis for comparison of urine-derived renal progenitors to public nephron progenitor data sets and cell lines used in this study and culture conditions, please see supplemental materials and methods.

### Statistics

All data are presented as arithmetic means ± standard error of mean. At least 3 independent experiments were used for the calculation of mean values. P values of < 0.05 were considered significant.

## Supplementary information

Supplementary Methods, Figures, and Tables.

Supplemental Table S4.

Supplemental Table S5.

Supplemental Table S6.

Supplemental Table S7.

Supplemental Table S8.

## Data Availability

All raw and processed data used in this study have been archived in NCBI gene expression omnibus under GEO accession number GSE128281. (https://www.ncbi.nlm.nih.gov/geo/query/acc.cgi?acc=GSE128281).
