# Supplementary material for: The FGF, TGFβ and WNT axis Modulate Self-renewal of Human SIX2+ Urine Derived Renal Progenitor Cells
Source: Sci Rep. 2020 Jan 20;10:739. doi: 10.1038/s41598-020-57723-2 (PMC6970988; doi:10.1038/s41598-020-57723-2)
Supplement: Supplementary file 1 — Supplementary Methods, Figures, and Tables. [file 41598_2020_57723_MOESM1_ESM.docx]

**The FGF, TGFβ and WNT axis Modulate Self-renewal of Human SIX2^+^ Urine Derived Renal Progenitor Cells**

Md Shaifur Rahman, Wasco Wruck, Lucas-Sebastian Spitzhorn, Lisa Nguyen, Martina Bohndorf, Soraia Martins, Fatima Asar, Audrey Ncube, Lars Erichsen, Nina Graffmann, James Adjaye^*^

Institute for Stem Cell Research and Regenerative Medicine, Medical Faculty, Heinrich Heine University Düsseldorf, 40225 Düsseldorf, Germany

Md Shaifur Rahman and Wasco Wruck shared first-authorship.

**^*^Correspondence**

Prof. Dr. James Adjaye

Email: james.adjaye@med.uni-duesseldorf.de

Phone: 0049 211 8108191

**Supplemental Materials and Methods**

**Microarray data analyses**

Total RNA (1μg) preparations were hybridized on the PrimeView Human Gene Expression Array (Affymetrix, Thermo Fisher Scientific) at the core facility Biomedizinisches Forschungszentrum (BMFZ) of the Heinrich Heine University Düsseldorf. The raw data was imported into the R/Bioconductor environment (Gentleman *et al.*, 2004) and further processed with the package affy (Gautier *et al.*, 2004) using background-correction, logarithmic (base 2) transformation and normalization with the Robust Multi-array Average (RMA) method. The heatmap.2 function from the gplots package (Warnes *et al.*, 2015) was applied for cluster analysis and to generate heatmaps using Pearson correlation as similarity measure. Gene expression was detected as previously described (Graffmann *et al.*, 2016) using a detection-p-value threshold of 0.05. Differential gene expression was determined via the p-value from the limma package (Smyth, 2004) which was adjusted for false discovery rate using the q value package (Storey, 2002). Thresholds of 1.33 and 0.75 were used for up-/down-regulation of ratios and 0.05 for p-values. Venn diagrams were generated with the VennDiagram package (Chen and Boutros, 2011). Subsets from the venn diagrams were used for follow-up GO and pathway analyses. Gene expression data will be available online at the National Center of Biotechnology Information (NCBI) Gene Expression Omnibus.

**Meta-analysis for comparison of urine-derived renal stem cells to public nephron progenitor data sets**

Datasets containing transcriptome data from kidney biopsies for the meta-analysis have been downloaded from NCBI GEO from the accession numbers GSE74450 (fetal kidney) (Da Sacco *et al.* 2017), GSE75949 (fetal kidney) (https://www.ncbi.nlm.nih.gov/geo/query/acc.cgi?acc=GSE75949) and GSE23911 (adult kidney) (Lindgren *et al.,* 2011). As these datasets employed for the meta-analysis were produced on different platforms including rnaSeq and microarray technologies the heterogeneity induced by the technical platforms has to be taken into account when interpreting the results. RNAseq and microarray measurements were condensed to symbols of expressed genes using threshold for the detection-p-values in the Affymetrix and Illumina microarrays and threshold for RPKM in the rnaSeq data. The data was imported into the R/Bioconductor environment and the datasets were compared based on the expressed genes. The GSE23911 datasets were generated on the Illumina Beadchip platform. To condense the Illumina data to gene symbols we used the “meanmax”-method taking the mean values of all experiments for all probes and afterwards the maximum of all probes mapping to the same gene symbol. A gene was considered expressed when the mean detection-p-value of the above determined probe was below the threshold of p=0.05. The datasets GSE74450 and GSE75949 were based on RNAseq data and provided in the RPKM format. Here, we applied a threshold of RPKM > 1 to determine gene expression using the “meanmax”-method described above to condense data to unique gene symbols. Expressed genes in UdRPCs were determined as described in the material and methods section of this article. A venn diagram was generated via the R package gplots (Warnes *et al*., 2015) comparing urine-derived renal progenitors with datasets from GSE74450, GSE75949 and GSE23911. Over-representation of GOs (for details see Methods section) was calculated for the intersection of all genesets and for the intersection of urine-derived renal progenitors with datasets from GSE74450 and GSE75949 and the intersection of urine-derived renal progenitors with datasets from GSE75949 only which had the highest overlaps in terms of gene numbers.

**KEGG pathway, GO and network analysis**

Gene ontology (GOs) terms were analysed within the Bioconductor environment employing the package GOstats (Falcon and Gentleman, 2007). GOs of category Biological Process (BP) were further summarized with the REVIGO tool (Supek *et al.*, 2011) to generate treemaps populating the parameter for allowed similarity with tiny=0.4. GO networks were generated from the REVIGO tool in xgmml format and imported into Cytoscape (Shannon *et al.*, 2003). To reduce the network to a readable size they were filtered in Cytoscape by the log10(p) between -3.75 and -2.75. The saturation of the red nodes representing GO terms indicates the significance via the p-value while the grey value of the edges represents their similarity. KEGG pathways (Kanehisa *et al.*, 2017) were downloaded from the KEGG server in March 2018 and tested for over-representation with the R-built-in hypergeometric test.

**Activated WNT pathway associated protein interaction network**

The network was constructed from the 20 most significantly up- and down down-regulated genes between CHIR99021 treatment and untreated controls. Genes were ranked by the limma-p-value and passed the criteria: detection p-value < 0.05 for the dedicated condition, ratio < 0.75 or ratio > 1.33, limma-p-value < 0.05. The resulting 40 genes are marked as green nodes in the network. Interacting proteins containing at least one protein coded by the 40 genes were retrieved from BioGrid version 3.4.161 (Chatr-Aryamontri *et al.*, 2017). To reduce complexity and increase visualization, the network was minimized by adding only the n=30 interacting proteins (marked as red nodes) with the most interactions to proteins coded by the 40 genes. The plot of the interactions network was drawn employing the R package network (Butts, 2008). Communities of related proteins within the network were detected employing an in-betweenness clustering analysis via the method cluster_edge_betweenness () from the R package igraph (Csardi and Nepusz, 2006).

**Effect of CHIR99021 stimulation on FGF-signaling**

To know the effect of CHIR99021 stimulation on FGF-signaling the gene expression in urine-derived renal progenitors treated with CHIR99021 compared to the same samples untreated using genes from the *FGF* and *FGFR* families and *BMP7* and *BMP4* from the *BMP* family. The heatmap.2 function from the gplots package (Warnes *et al.*, 2015) was applied to generate heatmaps using Pearson correlation as similarity measure.

**Culture supernatant analysis**

For the detection of cytokines secreted by the urine derived renal progenitors, we employed the Proteome Profiler Human Cytokine Array Panel A (R&D Systems, MA, USA) following the manufacturer's instructions and as described previously (Jungbluth, P. *et al.* 2019). 1.5 ml of conditioned medium from cultured UdRPCs at a density of 95% was used. The array was evaluated by detection of the emitted chemiluminescence. The pixel density of each spotted cytokine was analysed using the software ImageJ. All spots on the membrane including reference and negative control spots were measured separately. Correlation variations and *p* values were calculated based on the pixel density.

**Generation of iPSC from urine-derived renal progenitor cells**

Four distinct urine derived renal progenitor cell samples were reprogrammed into iPSCs (n=4, four lines) using an integration-free episomal based transfection system without pathway inhibition. Briefly, urine derived renal progenitor cells were nucleofected with two plasmids pEP4 E02S ET2K (Addgene plasmid #20927) and pEP4 E02S CK2M EN2L (Addgene plasmid #20924) expressing a combination of pluripotency factors including OCT4, SOX2, LIN28, c-MYC, KLF4, and NANOG using the Amaxa 4D-Nucleofector Kit according to the manufacturer’s guidelines and as described previously. The nucleofected cells were cultured on Matrigel coated 6-well plate containing StemMACs or mTeSR media under hypoxic conditions. Emerging colonies were picked and transferred to a new plate and cultured under normoxic conditions. After few passaging, vector-dilution PCR and genomic DNA fingerprinting were performed. Karyotyping was performed at the Institute of Human Genetics and Anthropology, Heinrich Heine University, Düsseldorf. Finally, embryoid body (EB) formation and analysis were carried out.

**Albumin endocytosis assay**

Urine derived renal cells were plated at a density of 40% without coating. After two days the cells were washed 1X with PBS and incubated in new medium contained 20 μg/ml of bovine serum albumin (BSA)-Alexa Fluor 488 conjugate (catalog no. A13100; Thermo Fischer) for 1 h at 37°C. Thereafter, the cells were washed three times with ice-cold PBS and fixed with 4% PFA for 15 min. Cell-associated fluorescence was analyzed using an excitation wavelength of 488 nm and an emission wavelength of 540 nm and imaged using a florescence microscope (LSM700; Zeiss, Oberkochen, Germany).

**Analysis of cell proliferation**

Cell proliferation were analysed using resazurin metabolic colorimetric assay. Urine derived renal progenitor cells were seeded (1x10^4^ cells/well) in a 6-well plate and incubated at 37°C in a humidified atmosphere at 5% CO_2_. The medium was substituted with 10% of a resazurin solution (0.1 mg/ml resazurin salt solution (Sigma- Aldrich) in PBS) with an end-volume of 2 ml per well and changed on daily basis. The cultures were incubated for 4h at 37°C in 5% CO_2_. Following this incubation period, the resazurin-containing medium was collected and the rate of resazurin conversion to resofurin by metabolically active cells was evaluated by spectrophotometric analysis at 570 and 600 nm. A final optical density (O.D.f) was determined for each sample, as follows: (O.D. 570/O.D.600)-(O.D.570c/O.D. c 600), where ‘OD.c’ are the O.D.s of control samples (fresh medium supplemented with resazurin, never in contact with cells). This procedure was carried out for 9 days, at the same hour, in triplicate.

**Bisulfite genomic sequencing**

Bisulfite sequencing was performed following bisulfite conversion with the EpiTec Kit (Qiagen, Hilden, Germany). Primers were designed after excluding pseudogenes or other closely related genomic sequences which could interfere with specific amplification by amplicon and primer sequences comparison in BLAT sequence database (https://genome.ucsc.edu/FAQ/FAQblat.html). In brief, the amplification conditions were denaturation at 95°C for 13min. followed by 37 cycles of 95°C for 50s, TM for 45s and 72°C for 30s. The amplification product is 469 bp in size. Amplification product was cloned into a pCR2.1 vector using the TA Cloning Kit (Invitrogen, Carlsbad, United States) according to the manufacturer’s instructions. On average 30 clones were sequenced using the BigDye Terminator Cycle Sequencing Kit (Applied Biosystems, Foster City, United States) on a DNA analyzer 3700 (Applied Biosystems) with M13 primer to obtain a representative methylation profile the OCT4 promoter region. 5´-regulatory gene sequences are defined by +1 transcription start of the following sequence: Homo sapiens POU class 5 homeobox 1 (POU5F1), transcript variant 1, mRNA. NCBI Reference Sequence: NM_002701.6. For SIX2 bisulfite sequencing we applied nested PCR by diltuing the pre-amplification in a ratio of 1:10 and used the second primer pair with the same PCR conditions for 37 additional cycles. For SIX2, NCBI Reference Sequence: NG_009360.1.

**Cell lines used in this study and culture condition**

The fibroblast cell used in this study were obtained from human foreskin fibroblast (HFF1) (ATCC, #ATCC-SCRC-1041, Manassas, VA, USA, www.atcc.org). Pluripotent stem cells (HFF-iPSCs (human foreskin fibroblast-derived induced pluripotent stem cells (iPSCs)) and ESCs (H1 (#WA01) and H9 (#WA09), WiCell Research Institute, Madison, WI, USA, www.wicell.org) were cultured in mTeSR on cell culture dishes coated with Matrigel (BD). Media were replaced change every day. Passaging of pluripotent stem cells was carried out with a splitting ratio of 1:3 to 1:10. Passaging was conducted manually using a syringe needle and a pipette under a binocular microscope or using a cell scraper and PBS (--).

**References**

Butts, C. network: A Package for Managing Relational Data in R. *Journal of Statistical Software* **24,** 01-36 (2008).

Chatr-Aryamontri, A. *et al.* The BioGRID interaction database: 2017 update. *Nucleic Acids Res.* **45,** 369–379 (2017).

Chen, H., Boutros, P.C. VennDiagram: a package for the generation of highly-customizable Venn and Euler diagrams in R. *BMC Bioinformatics* **12,** 35 (2011).

Csardi, G., Nepusz, T. The igraph software package for complex network research*. Inter. Journal Complex Systems* 1695 (2006).

Falcon, S., Gentleman, R. Using GOstats to test gene lists for GO term association. *Bioinforma. Oxf. Engl.* **23,** 257–258 (2007).

Gautier, L., Cope, L., Bolstad, B.M., Irizarry, R.A. affy--analysis of Affymetrix GeneChip data at the probe level. *Bioinforma. Oxf. Engl.* **20,** 307–315 (2004).

Gentleman, R.C. *et al.* Bioconductor: open software development for computational biology and bioinformatics. *Genome Biol.* **5,** R80 (2004).

Graffmann, N.*et al.* Modeling Nonalcoholic Fatty Liver Disease with Human Pluripotent Stem Cell-Derived Immature Hepatocyte-Like Cells Reveals Activation of PLIN2 and Confirms Regulatory Functions of Peroxisome Proliferator-Activated Receptor Alpha. *Stem Cells Dev.* 2**5,** 1119–1133 (2016).

Kanehisa, M., Furumichi, M., Tanabe, M., Sato, Y., Morishima, K. KEGG: new perspectives on genomes, pathways, diseases and drugs. *Nucleic Acids Res.* **45:** D353–D361 (2017).

Shannon, P. *et al.* Cytoscape: A Software Environment for Integrated Models of Biomolecular Interaction Networks. *Genome Res.* **13,** 2498–2504 (2003).

Smyth, G.K. Linear Models and Empirical Bayes Methods for Assessing Differential Expression in Microarray Experiments. *Stat. Appl. Genet. Mol. Biol.* 3 (2004). http://www.bepress.com/sagmb/vol3/iss1/art3 (Accessed October 28, 2011).

Storey, J.D. A direct approach to false discovery rates. *J. R. Stat. Soc. Ser. B. Stat. Methodol.* **64,** 479–498 (2002).

Supek, F., Bošnjak, M., Škunca, N., Šmuc, T. (2011). REVIGO Summarizes and Visualizes Long Lists of Gene Ontology Terms ed. C. Gibas. *PLoS ONE* **6,** e21800 (2002).

Warnes, G.R. *et al.* gplots: Various R Programming Tools for Plotting Data. (2015). <http://CRAN.R-project.org/package=gplots>.

Da Sacco, S. et al. Direct Isolation and Characterization of Human Nephron Progenitors: Characterization of Human Nephron Progenitors. *Stem Cells Translational Medicine.* **6,** 419–433 (2017).

https://www.ncbi.nlm.nih.gov/geo/query/acc.cgi?acc=GSE75949 (accessed on 23th October 2019)

Jungbluth, P. *et al.* Human iPSC-derived iMSCs improve bone regeneration in mini-pigs. *Bone Res*. **7**, 32 (2019) doi:10.1038/s41413-019-0069-4

Lindgren, D. et al. Isolation and characterization of progenitor-like cells from human renal proximal tubules. Am. *J. Pathol.* **178,** 828–837 (2011).

**Supplemental Figure S1**


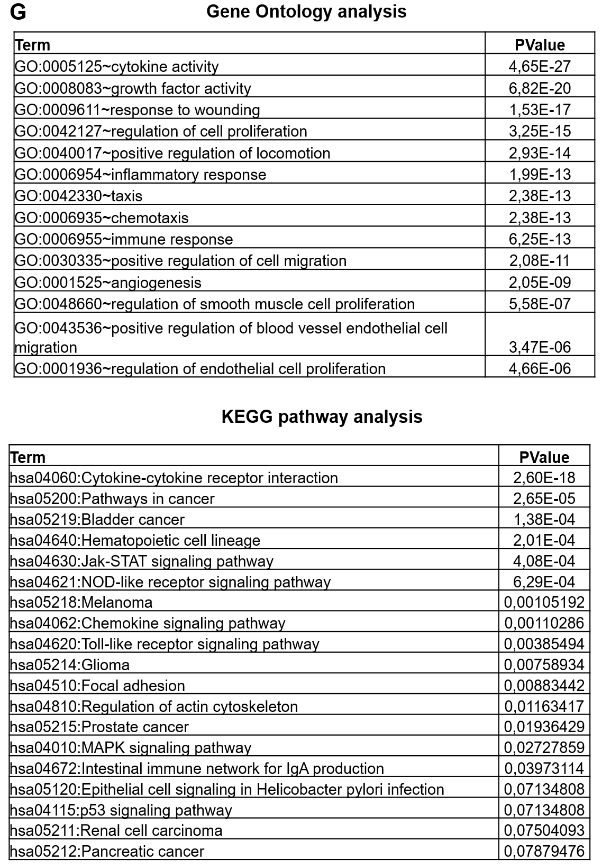

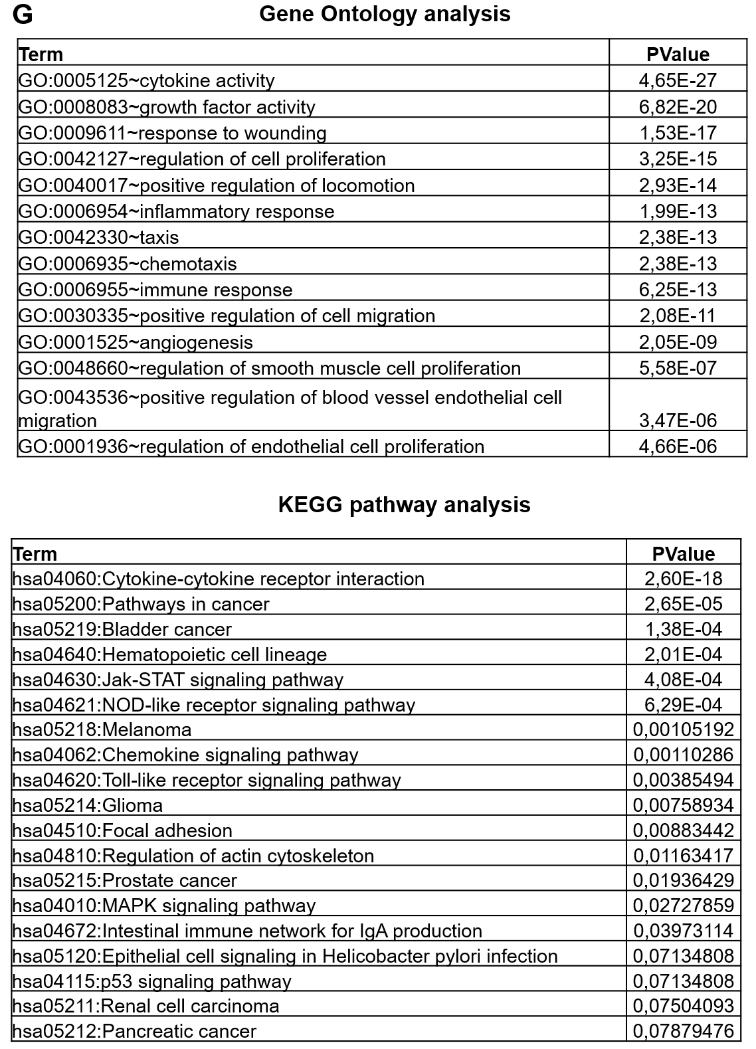

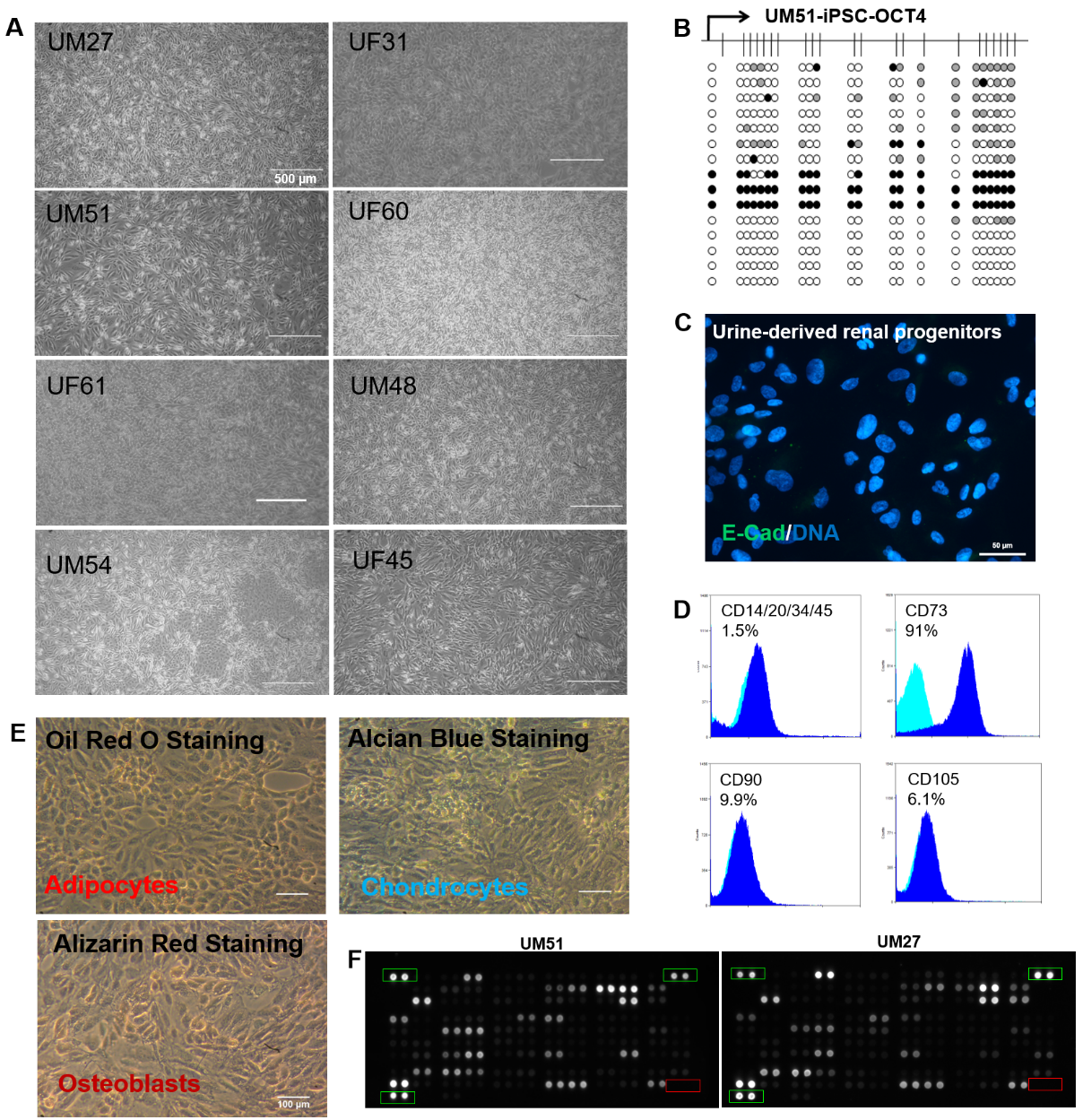


**Supplemental Figure S1** (A) The “rice grain’’ fibroblast-like morphology of the isolated cell samples (B) Detailed analysis by bisulfite sequencing of CpG island methylation patterns within the 5´- regulatory region of the OCT4 gene in UM51 (control) and its iPSC derivative. Detailed CpG methylation profiles of the OCT4 5´-regulatory region are documented as revealed by bisulfite sequencing. Filled circles (black) denote methylated CpG dinucleotides, white denote unmethylated CpGs and gray CpG dinucleotides of unknown methylation status. Arrows indicate the transcription start site. (C) Representative picture of E-Cad staining. (D) Immuno-phenotyping for MSC markers. (E) Control cell staining for *In Vitro* differentiation into Adipocytes, Chondrocytes and Osteoblasts. (F) Secretome profile membrane and (G) Secretome genes related GOs and KEGG Pathways.

**Supplemental Figure S2**

**Supplemental Figure S2** Venn diagram comparing expressed genes in urine-derived renal progenitor cells (UdRPCs) and kidney biopsy-derived datasets from GSE74450, GSE75949 and GSE23911. Urine-derived renal progenitor cells have high overlap with kidney biopsies (4411 gene subset) and among them particularly with the fetal kidney-derived ones GSE74450 and GSE75949 (2885 gene subset). Results of the GO overrepresentation analysis of selected intersection sets from the Venn diagram are shown in Supplemental Table S5.

**Supplemental Figure S3**


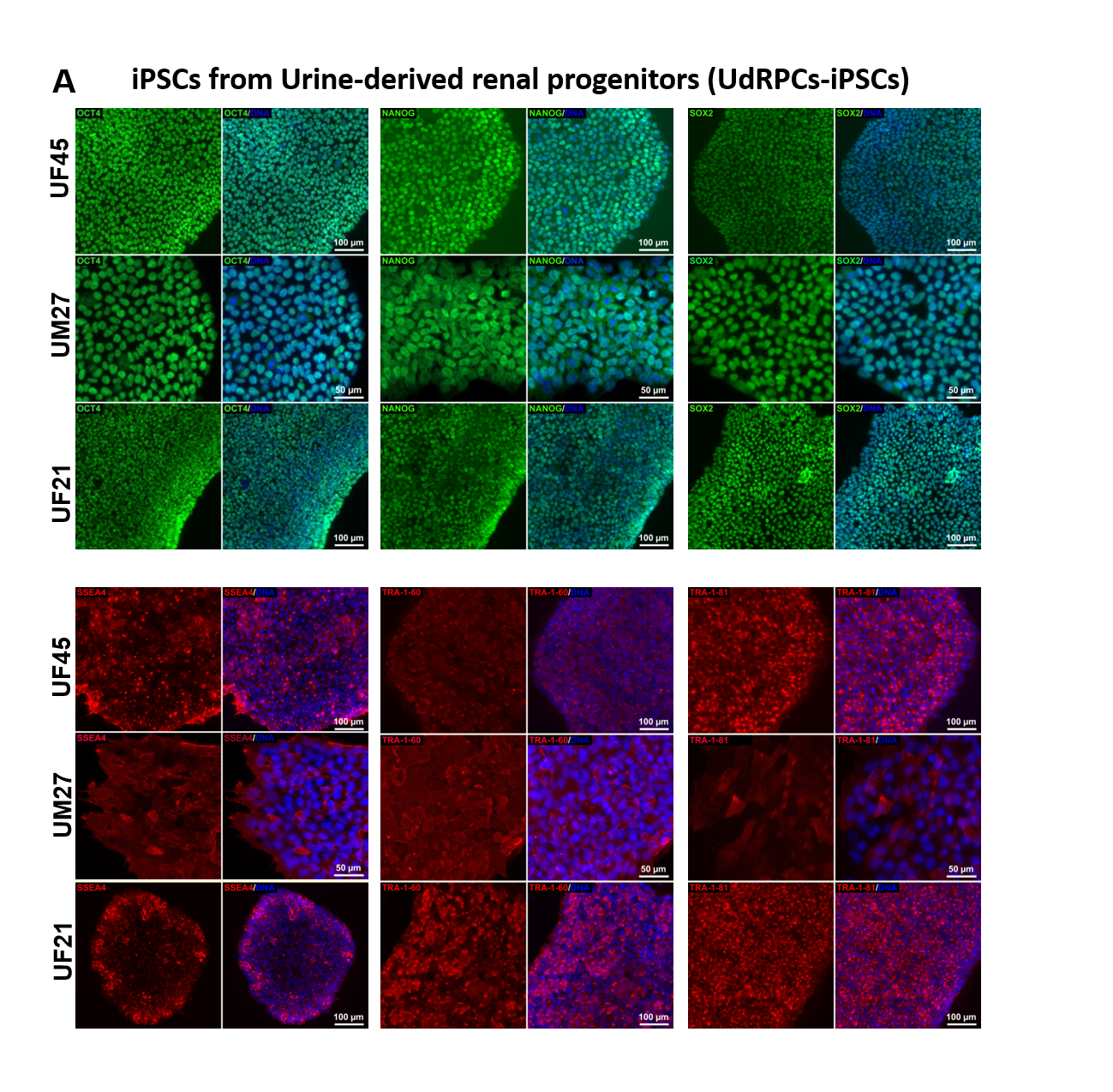


**
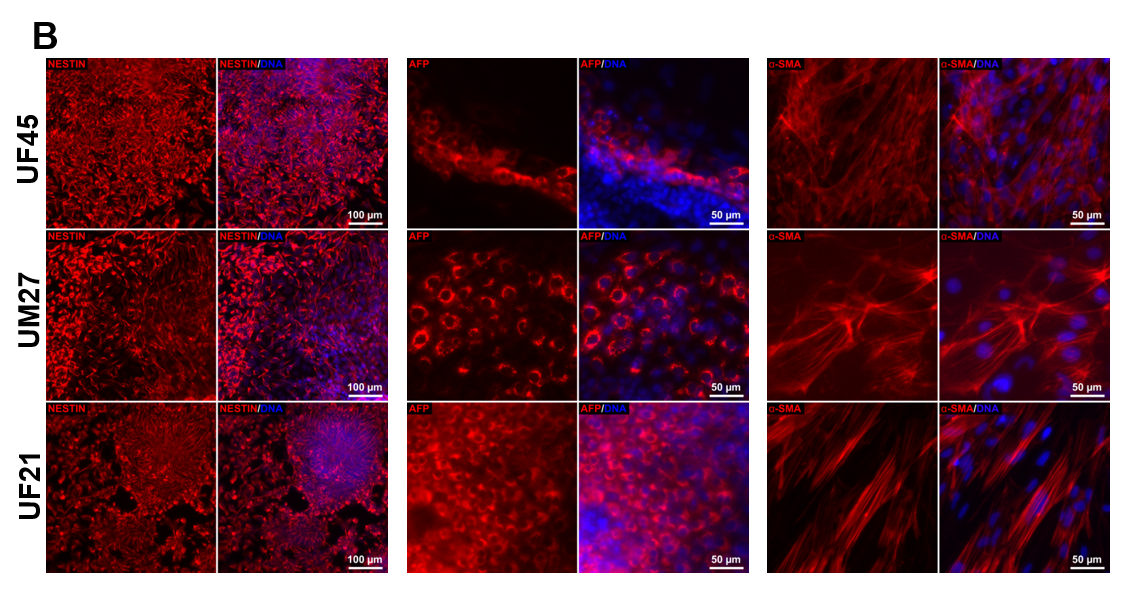
**

**
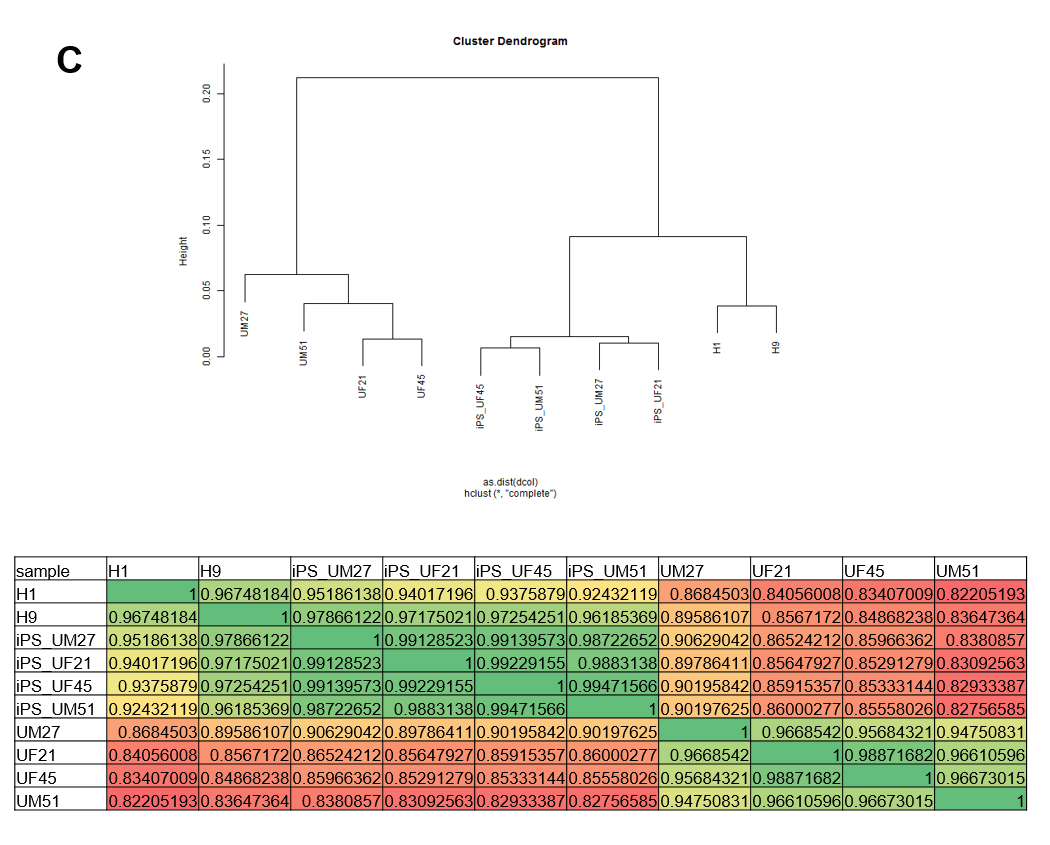
**

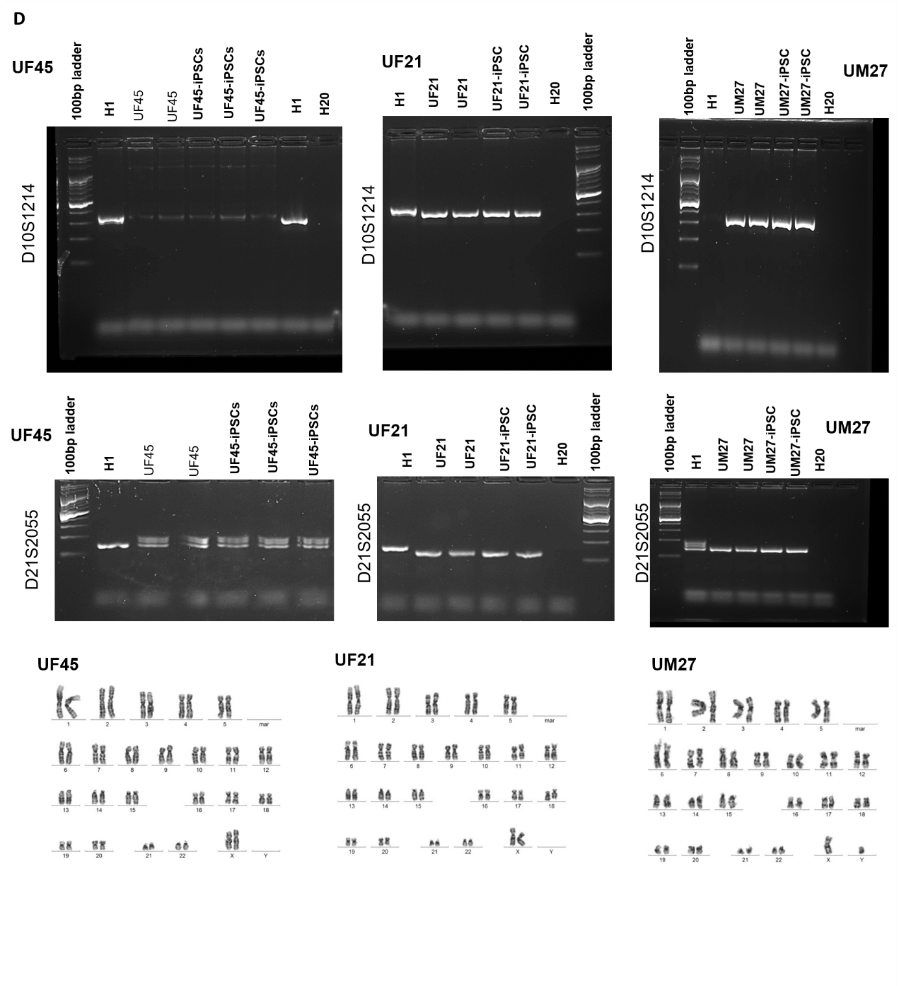


**Supplemental Figure S3** Generation and characterization of iPSCs from urine-derived renal progenitor cell (UdRPC-iPSCs). (A) The reprogrammed vector-free UdRPCs-iPSCs stained positive for the transcription factors Oct4, Sox2 and Nanog and for the surface markers TRA-1-60, TRA-1-81 and SSEA-4 confirmed by immunofluorescence. (B) The immunofluorescence-based study shows a successful undirected differentiation into the mesoderm lineage detected with the mesoderm marker α-SMA and a successful specification along the endoderm layer confirmed with AFP, as well as the ectoderm layer proved with Nestin. (C) Dendrogram resulting from hierarchical clustering of global gene expression profiles of UdRPCs-iPSCs, Urine-derived renal progenitor cells (UdRPCs), hREPCs, CHIR treated UdRPCS and established ESCs (H1, H9). Transcriptomes of UdRPCs-iPSCs cluster with H1, H9 while those of the UdRPCs cluster separately. Pearson correlation analysis of transcriptome data revealed a high correlation (green) of UdRPCs-iPSCs with ESCs but low correlation with UdRPCs. Pearson's correlation coefficient was calculated in which each replicate was pairwise compared with each other replicate. A value of 1 indicates perfect linear correlation while a value of 0 implies no correlation. (D) The origin of the formed iPSCs was assigned to its donor UdRPC line by determining the individual DNA signature. For this purpose, a PCR-based DNA fingerprinting using specific primer sets, which amplify different VNTRs (variable number of tandem repeats) was employed. Ultimately, the genotyping provide evidence, that reprogrammed iPSC clones originate from their parental UdRPC line and hence exclude the possibility of cross-contamination. Collecting all data, the excellent quality and integrity of the reprogrammed urinary progenitor cells was proven by a normal 46, XY karyotype.

**Supplemental Figure S4**

**
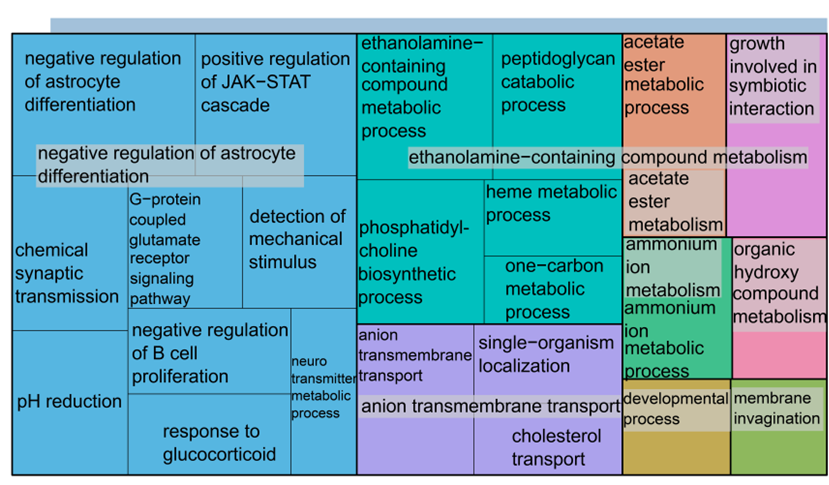
**

**Supplemental Figure S4** ESC-exclusively GOs when compared to HFF-iPSCs and iPSCs from urine-derived renal progenitors (UdRPC-iPSCs). Treemap summarizing the GO-BP terms overrepresented in the 197 genes expressed exclusively in ESCs. The largest most significant group is associated with “negative regulation of astrocyte differentiation”, second comes “anion transmembrane transport”.

**Supplemental Figure S5**

**
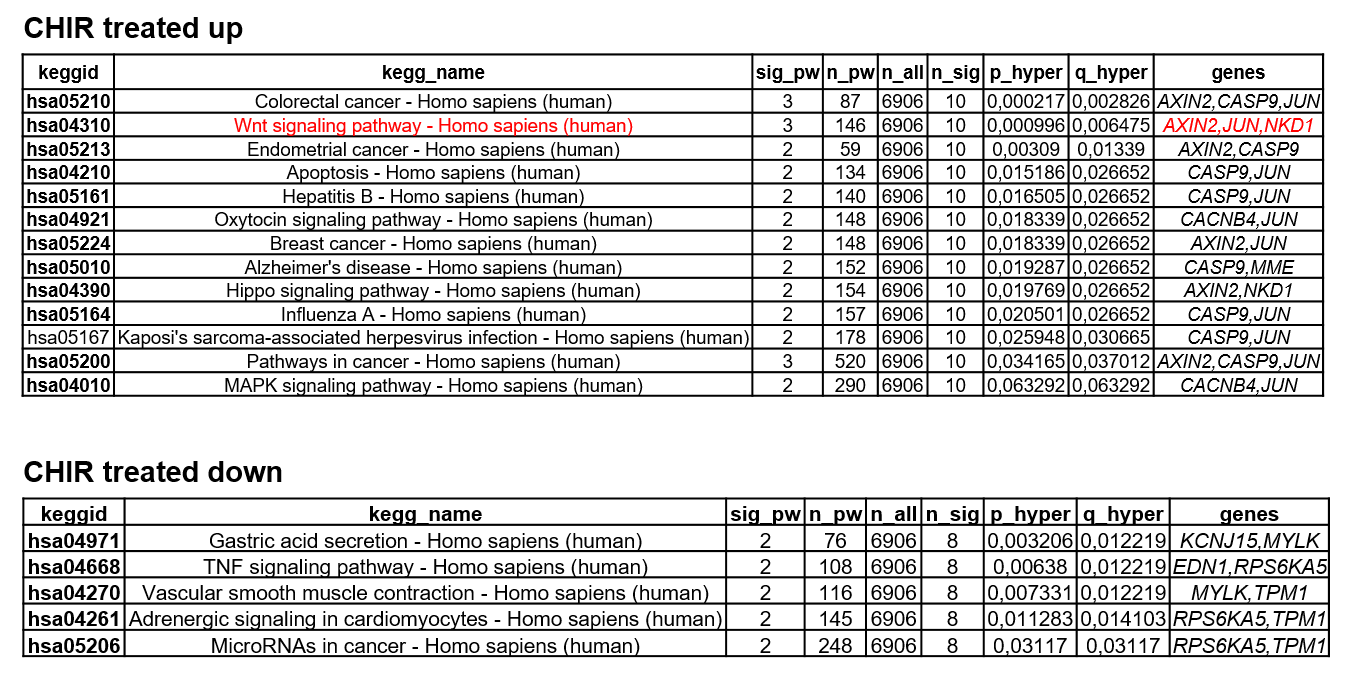
**

**Supplemental Figure S5** KEGG pathways associated with genes up and down regulated upon CHIR treatment of the urine-derived renal progenitors.

**Supplemental Figure S6**


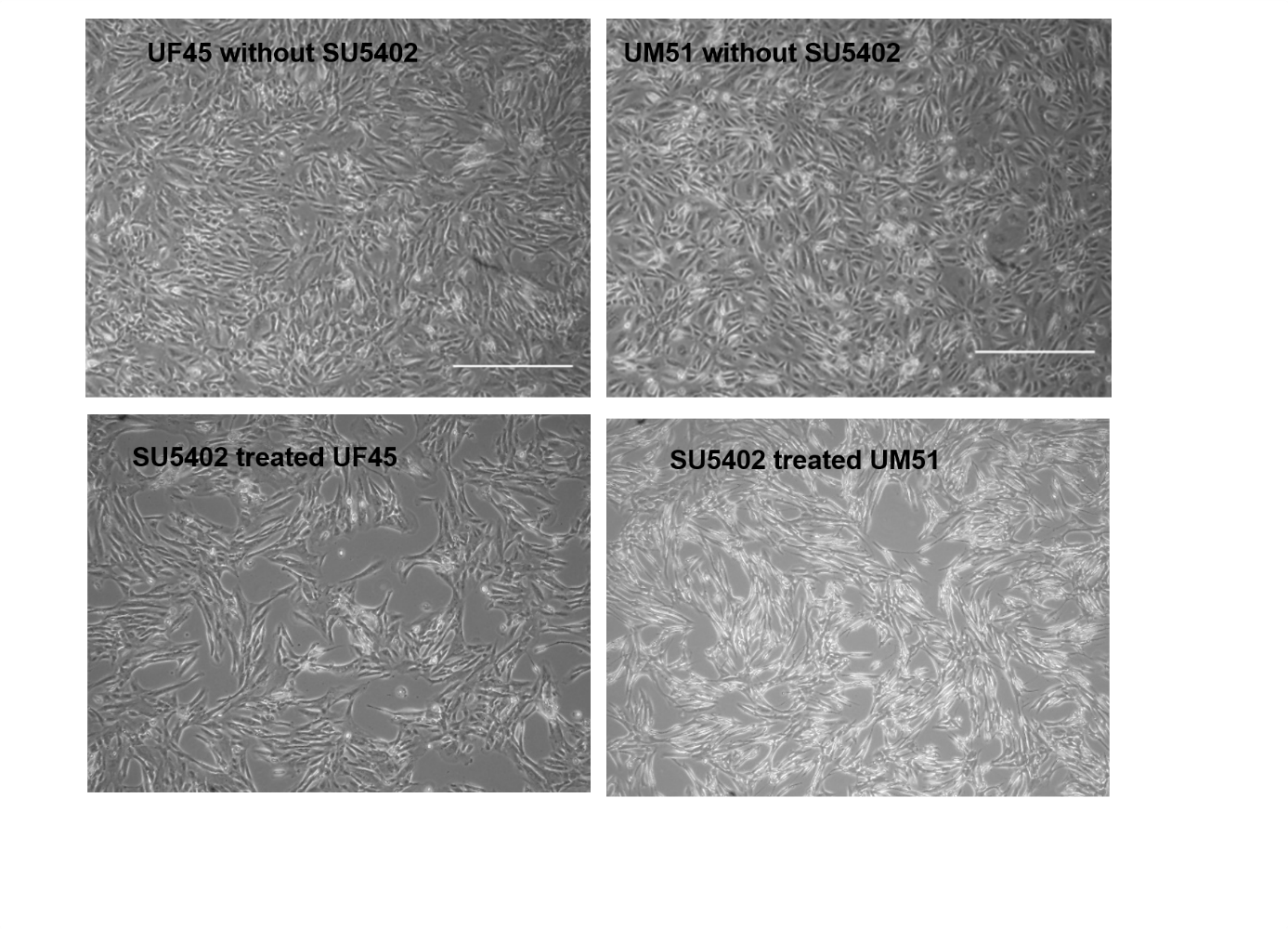


**Supplemental Figure S6** Changes in morphology and growth upon treatment with FGF receptor inhibitor small molecule SU5402 of the urine-derived renal progenitors.

**Supplemental Table S1** Urine-derived renal progenitor cell samples overview.

| **Sample ID** | **Gender** | **Age** | **Ethnicity** | **iPSCs** | **CYP2D6 Genotype** | **CYP2D6 Phenotype** |
| --- | --- | --- | --- | --- | --- | --- |
| **UM51** | Male | 51 | African | YES | CYP2D6*4/*17 | Intermidiate metabolizer (IM) |
| **UF45** | Female | 45 | Caucasian | YES | CYP2D6*1/*4 | Normal metabolizer (NM) |
| **UF31** | Female | 31 | African | YES | CYP2D6*1/*41 | Ultra-rapid metabolizer (UM) |
| **UF21** | Female | 21 | Caucasian | YES | CYP2D6*2/*2 | Normal metabolizer (NM) |
| **UM27** | Male | 27 | Caucasian | - | CYP2D6*1x2/*4 | Normal metabolizer (NM) |
| **UM54** | Male | 54 | Caucasian | - | - | - |
| **UF61** | Female | 61 | Caucasian | - | - | - |
| **UF27** | Female | 27 | Caucasian | - | - | - |
| **UM48** | Male | 48 | African | - | - | - |
| **UF60** | Female | 60 | Caucasian | - | - | - |

**UM: Urine Male and UF: Urine Female**

**Supplemental Table S2.** List of Antibodies used for immunocytochemistry/flow-cytometry.

| **Marker type** | **Antibody** | **Dilution** | **Supplier/ Origin company** |
| --- | --- | --- | --- |
| Renal progenitor marker | Anti SIX2 mAb | 1:100 | Abnova, Cat#H00010736-M01 |
| Pluripotency markers | CD133 | 1:500 | US Biological Cat# C2514-90B, RRID:AB_2284567Boster Biological; PA2049 |
| Pluripotency markers | Rabbit anti-OCT4 | 1:400 | Cell Signaling Technology Cat# 2840S, RRID:AB_2167691 |
| Pluripotency markers | Rabbit anti-SOX2 | 1:400 | Cell Signaling Technology Cat# 3579S, RRID:AB_2195767 |
| Pluripotency markers | Rabbit anti-NANOG | 1:800 | Cell Signaling Technology Cat# 4903S, RRID:AB_10559205 |
| Pluripotency markers | Mouse anti-SSEA4 | 1:1000 | Cell Signaling Technology Cat# 4755S, RRID:AB_1264259 |
| Differentiation markers | Mouse anti-SOX17 | 1:50 | R and D Systems Cat# AF1924, RRID:AB_355060 |
| Differentiation markers | Rabbit anti-AFP | 1:200 | Cell Signaling Technology Cat# 2137S, RRID:AB_2209744 |
| Differentiation markers | anti-Nestin | 1:250 | Sigma-Aldrich Cat# N5413, RRID:AB_1841032 |
| Differentiation markers | anti-aSMA | 1:1000 | Dako Cat# M0851, RRID:AB_2223500 |
| MSC Marker | Vimentin (5G3F10) mouse mAb | 1:200 | Cell Signaling Technology, USA, 3390 |
| Epithelial marker | E-Cad (24E10) rabbit | 1:200 | Cell Signaling Technology, USA |
| Renal Marker | Rabbit anti-CK19 | 1:100 | Novus Biologicals, NB100-687 |
| Pluripotency markers | C-Kit (H-300) rabbit polyclonal IgG | 1:200 | Tebu Bio, Germany |
| Renal progenitor Marker | Rb CITED1 | 1:160 | Invitrogen, PA5-40585 |
| Renal progenitor Marker | m Anti WT1 clone 6F-H2 | 1:200 | MD Millipore Corp., O5-753 |

**Supplemental Table S3.** List of primers

| Genes Name | Primer Sequences | Product  size (bp) |
| --- | --- | --- |
| *BMP7* | F1: CAACCTCGTGGAACATGACAAG, R1: AAGATCAAACCGGAACTCTCGAT | 70 |
| *CD133* | F1: GACTTGCGAACTCTCTTGAATGA, R1: GGTAGTGTTGTACTGGGCCAAT | 222 |
| *RPL37A* | F1: GTGGTTCCTGCATGAAGACAGTG, R1: TTCTGATGGCGGACTTTACCG | 84 |
| *OCT4* | F1: GAGGGAGAGAGGGGTTGAGTAGTTTT, R1:ACTCCAACTTCTCCTTCTCCAACTTC | 469 |
| *SIX2* | F1: GGTATTATGTTTATGTTGTTTAT, R1: AACTAATAACTCTCCAAAATCT,  R2: AAAACTAATAACTCTCCAAAAT | 232 |
| *SIX2 for qRT-PCR* | F1: TCCTGGTCCCTCCGTAT, R1: TAGGGGCAGATAGACCA | 62 |

**Supplemental Table S4 (Excel file_1).** Sheet 1: Subsets of Venn diagram comparison of urine-derived renal progenitors of sample UM51 vs. hREPCs. Sheet 2: Overrepresented GOs in the exclusive UM51 subset with 566 genes from the Venn diagram comparison of UM51 vs. hREPCs. Sheet 3: Overrepresented GOs in the exclusive hREPCs subset with 438 genes from the Venn diagram comparison of UM51 vs. hREPCs. Sheet 4: Overrepresented GOs in the up-regulated genes (limma-p-value < 0.05, ratio > 2) from the overlap subset of the Venn diagram comparison of UM51 vs. hREPCs. Sheet 5: Overrepresented GOs in the down-regulated genes (limma-p-value < 0.05, ratio < 0.5) from the overlap subset of the Venn diagram comparison of UM51 vs. hREPCs.

**Supplemental Table S5 (Excel file_2).** Results of the GO overrepresentation analysis of selected intersection sets from the Venn diagram. Sheet 1: GOs in intersection of all genesets (4411). Sheet 2: kidney and development-related GOs in intersection of urine-derived renal progenitors (UdRPCs) with GSE74450 and GSE75949 (2885). Sheet 3: Selected renal progenitor related GOs in intersection of UdRPCs with GSE74450 and GSE75949. Sheet 4: GOs in intersection of UdRPCs with GSE75949 (1900). Sheet 5: genes in the subsets of the Venn diagram.

**Supplemental Table S6 (Excel file_3).** Sheet 1: Subsets of venn diagram comparison of urine-derived renal progenitors (UdRPCs) vs. fibroblasts. Sheet 2: Overrepresented GOs in the exclusive UdRPC subset with 463 genes from the venn diagram comparison of UdRPCs vs. fibroblasts.

**Supplemental Table S7 (Excel file_4).** Sheet 1: Subsets of Venn diagram comparison of iPSCs derived from urine-derived renal progenitors (UdRPC_iPSCs), iPSCs derived from human foreskin fibroblasts (B4_HFF_iPSCs) and human embryonic stem cells (ESCs). Sheet 2: Overrepresented GOs in the exclusive UdRPC_iPSCs subset with 150 genes from the Venn diagram comparison of UdRPC_iPSCs, B4_HFF_iPSCs and ESCs. Sheet 3: Overrepresented GOs in the exclusive B4_HFF_iPSCs subset with 312 genes from the Venn diagram comparison of UdRPC_iPSCs, B4_HFF_iPSCs and ESCs. Sheet 4: Overrepresented GOs in the exclusive ESCs subset with 197 genes from the Venn diagram comparison of UdRPC_iPSCs, B4_HFF_iPSCs and ESCs.

**Supplemental Table S8 (Excel file_5).** Sheet 1: Subsets of Venn diagram comparison of urine-derived renal progenitors (UdRPCs) treated with CHIR99021 vs. untreated UdRPCs. Sheet 2: The set of 2491 up-regulated genes (p<0.05, ratio>1.33) from the Venn diagram intersection of UdRPCs treated with CHIR99021 vs. untreated UdRPCs. Sheet 3: The set of 2043 down-regulated genes (p<0.05, ratio<0.75) from the Venn diagram intersection of UdRPCs treated with CHIR99021 vs. untreated UdRPCs. Sheet 4: The set of 7255 not regulated genes (p>0.05, 0.75<ratio<1.33) from the Venn diagram intersection of UdRPCs treated with CHIR99021 vs. untreated UdRPCs. Sheet 5: Overrepresented KEGG pathways in the set of 2491 up-regulated genes from the Venn diagram intersection of UdRPCs treated with CHIR99021 vs. untreated UdRPCs. Sheet 6: Overrepresented KEGG pathways in the set of 2043 down-regulated genes from the Venn diagram intersection of UdRPCs treated with CHIR99021 vs. untreated UdRPCs. Sheet 7: Novel genes beginning with LOC (without published symbol) in the set of 2491 up-regulated genes (p<0.05, ratio>1.33) from the Venn diagram intersection of UdRPCs treated with CHIR99021 vs. untreated UdRPCs. Sheet 8: Novel genes beginning with LOC (without published symbol) in the set of 2043 down-regulated genes (p<0.05, ratio<0.75) from the Venn diagram intersection of UdRPCs treated with CHIR99021 vs. untreated UdRPCs. Sheet 9: Novel genes beginning with LOC (without published symbol) in the set of 7255 down-regulated genes (p>0.05, 0.75<ratio<1.33) from the Venn diagram intersection of UdRPCs treated with CHIR99021 vs. untreated UdRPCs.
